# Impairment of human neural crest cell migration by prolonged exposure to interferon-beta

**DOI:** 10.1007/s00204-017-1966-1

**Published:** 2017-04-01

**Authors:** Giorgia Pallocca, Johanna Nyffeler, Xenia Dolde, Marianna Grinberg, Gerhard Gstraunthaler, Tanja Waldmann, Jörg Rahnenführer, Agapios Sachinidis, Marcel Leist

**Affiliations:** 1 Department of In Vitro Toxicology and Biomedicine, University of Konstanz, Box 657, 78457 Konstanz, Germany; 2 Department of Statistics, TU Dortmund, 44139 Dortmund, Germany; 3 Department of Physiology, University of Innsbruck, 6020 Innsbruck, Austria; 4 Center of Physiology and Pathophysiology, Institute of Neurophysiology, University of Cologne, 50931 Cologne, Germany

**Keywords:** Interferons, Neural crest, Cell migration, JAK-STAT pathway, Developmental toxicity

## Abstract

Human cell-based toxicological assays have been used successfully to detect known toxicants, and to distinguish them from negative controls. However, there is at present little experience on how to deal with hits from screens of compounds with yet unknown hazard. As a case study to this issue, we characterized human interferon-beta (IFNβ) as potential developmental toxicant affecting neural crest cells (NCC). The protein was identified as a hit during a screen of clinically used drugs in the ‘migration inhibition of neural crest’ (MINC) assay. Concentration–response studies in the MINC combined with immunocytochemistry and mRNA quantification of cellular markers showed that IFNβ inhibited NCC migration at concentrations as low as 20 pM. The effective concentrations found here correspond to levels found in human plasma, and they were neither cytostatic nor cytotoxic nor did they did they affect the differentiation state and overall phenotype of NCC. Data from two other migration assays confirmed that picomolar concentration of IFNβ reduced the motility of NCC, while other interferons were less potent. The activation of JAK kinase by IFNβ, as suggested by bioinformatics analysis of the transcriptome changes, was confirmed by biochemical methods. The degree and duration of pathway activation correlated with the extent of migration inhibition, and pharmacological block of this signaling pathway before, or up to 6 h after exposure to the cytokine prevented the effects of IFNβ on migration. Thus, the reduction of vital functions of human NCC is a hitherto unknown potential hazard of endogenous or pharmacologically applied interferons.

**Electronic supplementary material:**

The online version of this article (doi:10.1007/s00204-017-1966-1) contains supplementary material, which is available to authorized users.

## Introduction

Any kind of external interference which alters the spatio-temporal organization of development can lead to developmental defects (Smirnova et al. 2014; Aschner et al. 2017). Where protein signals are involved in the fine-tuning of cell and organ development, antigenic properties and receptor affinities play an important role. As these factors are often species-specific, the developmental hazard, and many other side effects of cytokines, antibodies and blood factors are difficult to assess in animal models. As alternative approach, human cell-based test systems have been developed and they play an increasingly important role in drug development.

The approach of using in vitro tests is based on the strategy that a battery of assays may be assembled that covers the majority of fundamental developmental processes (Leist et al. 2014; Smirnova et al. 2014; Schmidt et al. 2016). These include, for instance, neural differentiation (Zimmer et al. 2011b; Balmer et al. 2012), neurite outgrowth (Stiegler et al. 2011; Krug et al. 2013a), gliogenesis (Fritsche et al. 2005; Kleiderman et al. 2016), myelination (Zurich et al. 2000, 2002), or synaptogenesis (Harrill et al. 2011).

In this context, the migration inhibition of neural crest cells (MINC) assay was developed as the first method to test effects of toxicants on the function of the neural crest (NC) during development (Zimmer et al. 2012; Dreser et al. 2015). The importance of this developmental stage justified the inclusion of the MINC assay in the in vitro test battery of the European research consortium ESNATS (Embryonic Stem cell-based Novel Alternative Testing Strategies) (Zimmer et al. 2014). The NC is a multipotent migratory cell population that emerges from the dorsal aspect of the neural tube in the early phases of development. NC cells (NCC) are capable of long range migration, which is finely regulated in the embryo. During migration, and at the final destination, NCC give rise to a multitude of different cell types, supporting the formation of cartilage and bone of the face, but also peripheral and enteric neurons, melanocytes, and some cardiomyocytes (Huang and Saint-Jeannet 2004).

A large percentage of developmental disorders (e.g., congenital heart defects, oro-facial clefts, and Hirschsprung’s disease) are caused by NCC deficits (Simões-Costa and Bronner 2013), and these often correlate with neural tube defects. Such alterations can be induced by genetic factors (Lee et al. 2009) or exposure to pharmaceuticals (e.g., valproic acid) (Fuller et al. 2002) and pesticides (e.g., triadimefon) (Menegola et al. 2000).

The MINC assay has been used to screen and evaluate the effects of many different compounds on neural crest function (Zimmer et al. 2012, 2014; Dreser et al. 2015). In addition to known environmental toxicants, the screening identified some drug-like compounds as hits, and these included the type I interferon IFNβ (Zimmer et al. 2014). The developmental toxicity of IFNβ has been evaluated in cynomolgus monkeys, where an increased incidence of both abortions and stillbirths was observed (FDA 1999). Furthermore, epidemiological studies indicated that exposure to IFNβ in pregnancy is associated with lower mean birth weight, and preterm birth (Amato et al. 2010). For these reasons, women with MS are typically advised to discontinue the treatment before conceiving. IFNβ is classified as risk class C drug by the FDA, indicating that “animal reproduction studies have shown an adverse effect on the fetus and there are no adequate and well-controlled studies in humans, but potential benefits may warrant the use of the drug in pregnant women despite the potential risks” (Lu et al. 2012; Pozzilli and Pugliatti 2015).

The interferon family is clinically used for different purposes, as interferons have a crucial role in the anti-viral cell response, and in modulating several functions of the immune system. Type I interferons comprise IFNα and IFNβ. All type I IFNs bind the interferon alpha and beta receptor (IFNAR) subunits and produce structurally highly similar receptor-ligand complexes (Schreiber and Piehler 2015). While IFNα is mainly used for treatment of certain types of leukemia and hepatitis virus infections, the immunomodulatory drug IFNβ has proven effective in the treatment of relapsing-remitting multiple sclerosis (MS) (Dhib-Jalbut and Marks 2010). The mode of action of the latter cytokine is still not fully understood, but it induces an anti-inflammatory cytokine shift and prevents T-cell adhesion and extravasation across the blood–brain barrier. The type II interferon IFNγ binds to different receptors and has pronounced inflammatory and immune-stimulating properties (Schroder et al. 2004).

While elaborate strategies have been established for hit follow-up of drug discovery screens, such concepts still need to be developed for toxicological high-throughput testing. For statistical, strategic, and technical reasons, large screens have a high propensity to yield false-positives. Two approaches have proven useful in drug discovery: (1) follow-up of screen results under non-screen conditions (new compounds, new cells, low throughput, and low time constraint to allow stringent controls of each step), first with the original assay, then with secondary and tertiary tests; (2) exploration around the hit findings (use of related compounds; exploration of mechanistic and temporal consistency) to provide biological plausibility and a more robust data base. Such a strategy was explored in the present study to further substantiate or refute a potential developmental toxicity hazard of IFNβ. The adverse effects of IFNβ on NCC migration and proliferation were studied in three different assays. Additional analysis of transcriptome changes was performed, and the JAK-STAT signaling pathway was considered as a mediator of toxicity. The role of the pathway in a potential hazard of IFNβ was confirmed by detailed measurements of interferon effects on signaling and cell function in the presence of specific kinase inhibitors.

## Materials and methods

### Cell culture and neural crest differentiation

The reporter human embryonic stem cell line H9-Dll1 (GFP under Dll1 promoter) was provided by Mark Tomishima from the Memorial Sloan Kettering Cancer Centre (MSKCC, NY, USA). Import of the cells and all experiments was carried out according to German legislation under the license number 1710-79-1-4-27 of the Robert-Koch Institute.

H9-Dll1 cells were maintained on mouse embryonic fibroblasts in DMEM/F12 (Gibco™, Carlsbad, CA, USA) medium containing 20% of serum replacement (Knock-out SR, Gibco™), HEPES (Gibco™), L-gluthamine (Glutamax, Gibco™), non-essential amino acids (MEM NEAA, Gibco™), beta-mercaptoethanol (Gibco™), and basic fibroblast growth factor (10 ng/ml, Invitrogen™, Carlsbad, CA, USA).

Differentiation of hESC into NCC was initiated on mitomycin C (Sigma–Aldrich©, St. Louis, MO, USA) treated murine bone-marrow derived stromal MS5 cells and continued in N2 medium exactly as described previously (Zimmer et al. 2012).

The human breast adenocarcinoma cell line MDA-MB-231 (ATCC HTB-26™) was maintained in DMEM Glutamax (Gibco™) containing 10% FCS (Invitrogen) and 1% penicillin/streptomycin.

### Cell culture quality control

Cell culture experiments were performed according to GCCP (good cell culture practice) principles. All cell lines were regularly tested for mycoplasma contamination by the use of commercial kits (Venor Gem, Minerva Biolabs).

Identity of the used cell types was confirmed by STR (Short Tandem Repeat) analysis. Briefly, DNA samples from the H9 (NIH registry name WA09) and H9-Dll1 cell lines were prepared using a commercial kit (Puregene Cell Kit, Qiagen). The kit GlobalFiler^®^ PCR Amplification Kit (Thermofisher) was then used to determine the cell-specific profile for 16 different genomic loci using an Applied Biosystems GeneMapper Device. STR results showed 100% identity of the profiles of H9 and H9-Dll1, and these matched 100% the literature reference data (Josephson et al. 2006) (suppl. Fig S1).

### Interferon exposure during NCC migration

NCC were exposed for 48 h to non-cytotoxic concentration of different interferons (recombinant human IFN-beta 1a, recombinant human IFN-alpha 2a, recombinant human IFN-gamma) in N2 medium containing human EGF (20 ng/ml) and FGF2 (20 ng/ml) (all from R&D Systems GmbH^®^, Minneapolis, MN, USA). Interferon stock solutions were prepared in 0.1% BSA in PBS at concentrations of 200 nM and 10 µM, respectively, for IFN-beta 1a and IFN-alpha 2a. We assumed the nominal drug concentrations indicated in the figures/tables to correspond closely to the actual free concentrations, as protein binding of IFNβ is low (<10%). However, some absorption to the cell culture plastic might have occurred. Where indicated, cytosine arabinoside (AraC, Sigma) or JAK inhibitors (Ruxolitinib and Tofacitinib, Selleckchem©, Munich, Germany) were added. The migration assay was performed as described previously (Nyffeler et al. 2016). Briefly, NCC cells were seeded (95,000 cells/cm^2^) in 96-well plates (Corning©, NY, USA) previously coated with 10 µg/ml poly-l-ornithine in 100 µl phosphate buffered saline (PBS) (GE Healthcare Bio-Sciences©, Pittsburgh, PA, USA) and 1 µg/ml fibronectin and 1 µg/ml laminin (Sigma–Aldrich) in 100 µl PBS. Cells were seeded in the presence of silicon stoppers (Platypus Technologies, Madison, WI, USA) to create a circular cell-free area. One day after seeding, migration into the cell-free area was initiated by manual removal of the stoppers and the medium was replaced with medium containing the test compounds. After 48 h, NCC were stained with 1 µg/ml H-33342 (nuclei) and 533 nM calcein-AM (to stain the outline of viable cells) (Sigma–Aldrich) and imaged 30 min later on a high content imaging microscope (Cellomics ArrayScanVTI, Thermo Fischer©, Boston, MA, USA).

Viability was defined as the number of H-33342 and calcein double-positive cells, as determined by an automated algorithm described earlier (Stiegler et al. 2011; Krug et al. 2013b). For quantification of migration, a software tool (freely accessible at http://invitrotox.uni-konstanz.de/) was developed to estimate the most likely position of the previously cell-free area (covered by the silicon stopper), to set thresholds for color intensity for both dyes, and to count the number of H-33342 and calcein double-positive cells in the region of interest.

### EdU staining

Cells were treated for 48 h with 10 µM 5-ethynyl-deoxyuridine (EdU). Proliferating cells incorporate EdU and can be detected using a click reaction procedure, as described in the manufacturer’s protocol (EdU-Click 555, PanaTecs, Heilbronn, Germany). Images were acquired using a high content imaging microscope (Cell Insight Personal Imager, Thermo Fisher©). Proliferation was defined as the percentage of EdU-positive nuclei among all H-33342 positive nuclei.

### Immunofluorescence staining

Cells were seeded in Lumox^®^ multiwell plates (95,000 cells/cm^2^). After 42-h treatment, the middle of the well was scratched with a pipette tip to create a cell-free space in the well and cells were allowed to migrate for 6 h in presence of the treatment. Finally, the cells were fixed in 4% PFA for immunofluorescence staining. Cells were permeabilized for 10 min in 0.2% Triton and blocked with 10% FBS for 1 h. Afterwards, cells were incubated overnight with primary antibody (GM130, Abcam©, Cambridge, UK; TOM20, Santa Cruz Biotechnology™^,^ Santa Cruz, CA, USA) or Alexa Fluor 555 phalloidin diluted in 4% FBS. After a washing step, the secondary antibody was applied for 1 h. Cell nuclei were finally stained with H-33342. Images were acquired mainly from the scratch area (to capture migrating cells at a density typically also found in the standard cMINC assay setup) using a point laser scanning confocal microscope Zeiss LSM 700 (Zeiss©, Oberkochen, Germany).

### Western blot

Cells were seeded in 6-well plates (50,000 cells/cm^2^); after 24 h, cells were treated for the indicated times. Cells were then harvested in Laemmli buffer, boiled for 5 min at 95 °C and purified with the NucleoSpin Filters (Macherey–Nagel GmbH, Düren, Germany). Samples were run on SDS–PAGE. Transfer on nitrocellulose membranes was performed by using iBlot™ 2 Dry Blotting System (Invitrogen). Membranes were then incubated in 5% milk in T-TBS for 1 h and overnight with the primary antibody in 5% BSA in T-TBS at 4 °C (p-STAT1 Y701, Cell signaling technology). After washing steps, membranes were incubated with secondary antibody conjugated with horseradish peroxidase (GE Healthcare Bio-Sciences©) for 1 h at room temperature. Signal was finally detected using Pierce ECL wester blotting substrate (Thermo Scientific© Boston, MA, USA) and imaged with a Fusion-SL 3500 WL device and Fusion software (Bio-Rad™, Hercules, CA, USA). Afterwards, the membrane was further incubated for 3 h with the primary antibody specific for the housekeeping protein GAPDH (Invitrogen). The same procedure as described above was followed for detection and imaging of the housekeeping signal.

### NFkB translocation

Cells were stimulated by a cytokine mix (CM) containing 10 ng/ml tumor necrosis factor α (TNFα), 10 ng/ml interleukin 1β (IL-1 β), and 20 ng/mL IFNγ (R&D Systems, Wiesbaden, Germany) or with 500 pM IFNβ for 1 h. For NFkB measurement, cells were fixed, permeabilized, and stained with NFkB antibody (Santa Cruz Biotechnology™). NFkB translocation was measured with the high-throughput device CellInsight TM CX5 High Content Screening (Thermo Scientific©) using the nuclear translocation algorithm as described previously (Henn et al. 2011).

### Cell tracking

Live cell imaging was performed using an Axio Observer. Z1 microscope (Zeiss©) equipped with an incubation system (37 °C, 5% CO_2_) and the software Zen2. Cells were imaged for a period of 48 h, taking phase contrast pictures every 15 min with a 5x objective. Images of the last 30 h migration period were loaded in Fiji ImageJ and manually tracked with the plugin “Manual track” Schindelin et al. (2012). The tracked positions were then loaded into the freely available “Chemotaxis and Migration Tool” (Ibidi) to calculate the total distance and the cell speed as well as to create the track pictures.

### Transwell assay

Cells were treated for 42 h and then detached by using Accutase (Corning©), counted and seeded (50,000 cells/transwell) in upper chamber of previously coated Transwell Permeable Support plates (0.8 µm polycarbonate membrane, Costar, Corning©) in normal medium with addition of IFNβ (500 pM) or respective control. Ten percent FBS in normal medium was added in the lower chamber of the transwell. After 6 h of incubation at 37 °C, cells at the upper side of the membrane were removed and the cells attached to the lower side of the membrane were fixed and stained with crystal violet for 30 min, and then washed in current water and let dry. Cells were imaged with light microscope Axio Observer.Z1 microscope (Zeiss©) using the software PALM RoboSoftware (4 fields per condition, 20× magnification) and manually counted in ImageJ.

### Affymetrix gene chip analysis

Samples of ≥5 × 10^6^ cells were collected using RNA protect reagent from Qiagen. The RNA was quantified using a NanoDrop N-1000 spectrophotometer (NanoDrop, Wilmington, DE, USA), and the integrity of RNA was confirmed with a standard sense automated gel electrophoresis system (Experion, Bio-Rad™, Hercules, CA, USA). Analysis was then performed as described earlier (Krug et al. 2013b) using Affymetrix chip-based DNA microarray (Human genome U133 plus 2.0 arrays) with all standard quality control procedures. The raw CEL files and secondary Affymetrix chip data have been deposited in the GEO database according to the MIME rules, with the accession number GSE94521. The differentially expressed probe sets for each compound including fold changes and *p* values of the limma *t* test are given in supplementary tables provided in an Excel file format (supplemental Table 1; Fig S3).

### Biostatistics

The microarray data analysis (extrapolation and normalization of the array sets) was performed using the statistical programming language R (version 3.1.1) as described previously (Waldmann et al. 2014). For the normalization of the entire set of Affymetrix gene expression arrays, the Extrapolation Strategy (RMA+) algorithm (Harbron et al. 2007) was used that applies background correction, log_2_ transformation, quantile normalization, and a linear model fit to the normalized data to obtain a value for each probe set (PS) on each array. As reference, the normalization parameters obtained in earlier analyzes (Krug et al. 2013b) were used. After normalization, the difference between gene expression and corresponding controls was calculated (paired design). Differential expression was calculated using the R package ‘limma’ (Smyth et al. 2005). Here, the combined information of the complete set of genes is used by an empirical Bayes adjustment of the variance estimates of single genes. This form of a moderated *t* test is abbreviated here as ‘Limma *t* test’. The resulting *p* values were multiplicity-adjusted to control the false discovery rate (FDR) by the Benjamini–Hochberg procedure (Benjamini 1995). As a result, for each compound, a gene list was obtained, with corresponding estimates for log-fold changes and *p* values of the Limma t test (unadjusted and FDR adjusted).

Transcripts with FDR adjusted *p* values of ≤0.05 and fold change values of ≥1.8 or ≤0.55 were considered significantly deregulated and defined as differential expressed genes (DEG).

### Data display: heat map and principal component analysis

The software R (version 3.1.1), was used for all calculations and display of principal component analysis (PCA) and heatmaps. PCA plots were used to visualize expression data in two dimensions, representing the first two principal components. The percentages of the variances covered are indicated in the figures.

### Gene ontology (GO) and KEGG pathway enrichment analysis

The gene ontology group enrichment was performed using R (version 3.1.1) with the topGO package (Alexa et al. 2006) using Fisher’s exact test, and only results from the biological process ontology were kept. Here, again, the resulting *p* values were corrected for multiple testing by the method of Benjamini–Hochberg (Benjamini 1995).

The KEGG pathway analysis was performed using the R package “hgu133plus2.db” (Carlson 2015). Probesets were mapped to the identifiers used by KEGG for pathways in which the genes represented by the probesets are involved. The enrichment was then performed analogous to the gene ontology group enrichment using Fisher’s exact test.

Up- and down-regulated differentially expressed genes were analyzed separately for each treatment. Only GO classes and KEGG pathways with a BH (Benjamini–Hochberg)-adj. *p* values ≤0.05 were considered significant.

### GO superordinate classes distribution

Enriched GOs were then assigned to superordinate cell biological processes as already described previously (Waldmann et al. 2014) and distributed in six classes: migration/adhesion, metabolism, differentiation, signaling, stress response, and others. The migration class includes migration and adhesion-related-GO classes; stress response class includes cell death-, extracellular stressor-, and inflammation/immunity-related GO classes; signaling class consists of cell receptor activity-, second messenger (cAMP, cGMP, Ca2+) metabolism-, and kinase modification-related GO classes. Metabolism class comprises all GO classes covering metabolism activity; differentiation class includes cell differentiation related- GO classes; and; “other” class covers the others not otherwise classified GO classes.

### Quantitative PCR (qPCR) analysis

Cells were seeded in 6-well plates (50,000 cells/cm²); after 24 h, cells were treated for 48 h. Cells were then harvested and lysed in PeqGOLD Trifast™ (PEQlab©, Erlangen, Germany). Total RNA was isolated by phenol–chloroform extraction. Reverse transcription was performed using 1 µg RNA and the i-Script™ Reverse Transcription Supermix (Bio-Rad™) according to the manufacturer’s protocol. Quantitative real-time PCR was performed with SsoFast EvaGreen Supermix (Bio-Rad™) using a CFX96 Real-Time PCR Detection System (Bio-Rad™). The primer sequences are reported in the supplementary material (suppl. Fig S2). The cycle threshold values were determined using the Bio-Rad CFX Manager Software v2.0 (Bio-Rad™). Results were analyzed using the ΔCt method (Livak and Schmittgen 2001) and normalization for housekeeping genes exactly as described earlier (Zimmer et al. 2011a, b; Weng et al. 2012, 2014).

## Results

### Specific effects of IFNβ on human neural crest cell (NCC) migration

**Fig. 1:**
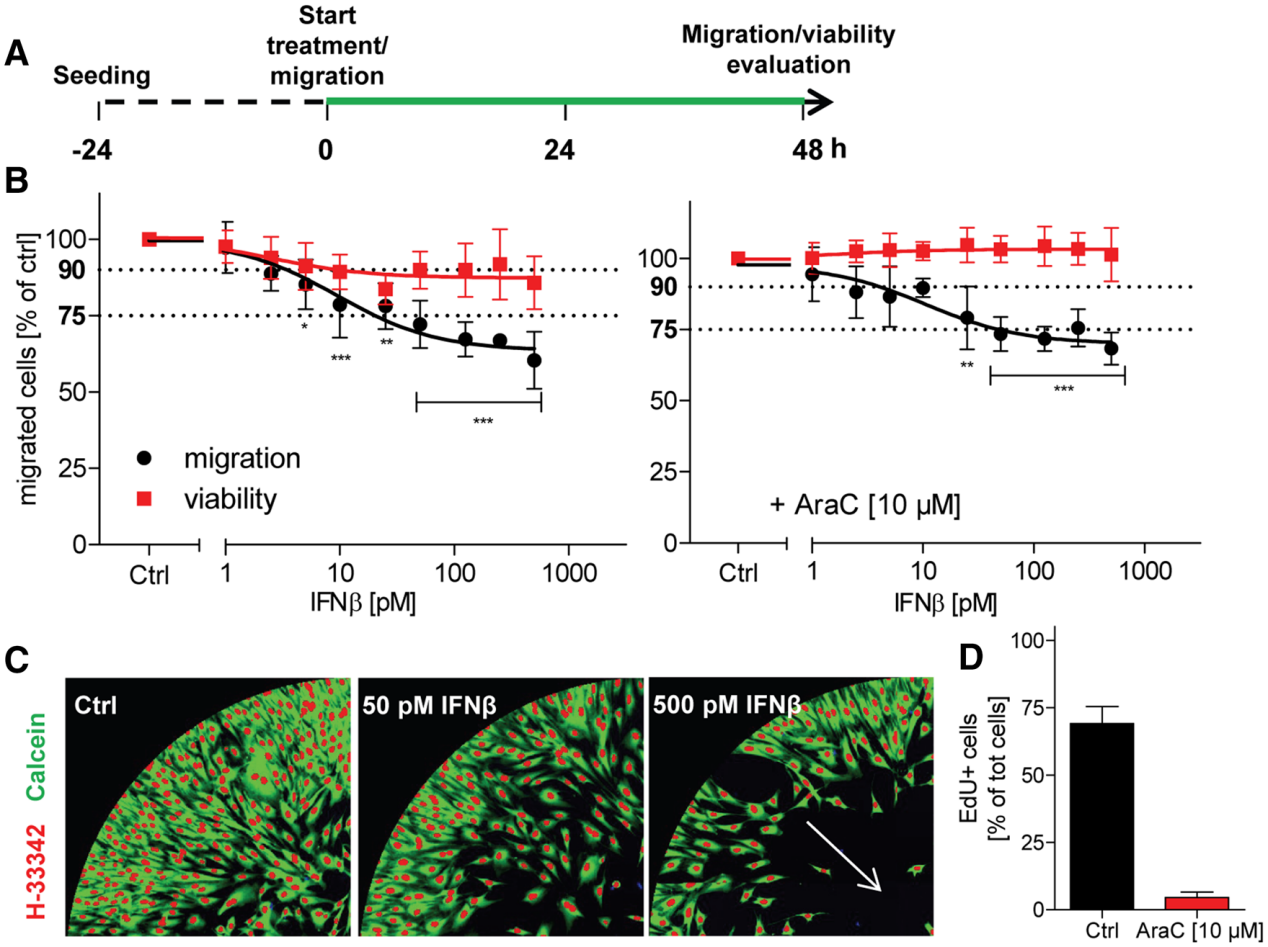
Impaired migration of NCC in the presence of IFNβ. **a** NCC were allowed to attach and recover for 24 h. Then, migration was started and, 48 h later, the number of migrated cells and the viability of the cell population were quantified. In standard experiments, NCC were exposed to IFNβ (marked in *green*) for the entire migration period. **b** NCC were exposed to IFNβ at the indicated concentrations. Cell viability and the number of migrated cells are expressed relative to control cells (Ctrl, 0.1% BSA in PBS). In one series of experiments (*right graph*), the cell culture medium used for all conditions was supplemented with the mitosis inhibitor cytosine arabinoside (AraC, 10 µM). Data are from three independent experiments. *Error bars* indicate standard deviations (SD). Statistics was performed for each endpoint by ANOVA, followed by Dunnet’s post-hoc test (*p ≤ 0.05, **p ≤ 0.01, ***p ≤ 0.001). Viability was considered to be impaired when it dropped below 90%; migration was considered to be impaired below 75%, compared to control (*dotted lines* at 75 and 90% are indicated for visual support). **c** Representative pictures of different migration assay exposure scenarios, taken at time 48 h. Nuclei are depicted in *red* (H-33342), while viable cells are shown in *green* (calcein). **d** NCC were exposed to culture medium supplemented with 5-ethynyl-2′-deoxyuridine (EdU, 10 µM) and they were treated with AraC (10 µM) or the respective control (Ctrl) for 48 h. Then, cells were stained with H-33342 (nuclei), and EdU-positive cells (EdU+) were quantified. Cell proliferation was expressed as percentage of EdU + cells out of the total number of cell nuclei. (Color figure online)

In a previous study (Zimmer et al. 2014), IFNβ was found as a positive hit in a screen of drugs for potential developmental toxicity in human NCC. To confirm the effects of the cytokine IFNβ on human NCC migration, a new automated and operator-independent test method (Nyffeler et al. 2016) was used. NCC, generated from pluripotent stem cells, were allowed to migrate in the presence or absence of IFNβ for 48 h (Fig. 1a). Picomolar concentrations of IFNβ induced a significant inhibition of migration (Fig. 1b, left graph), as indicated by the reduced number of cells found in the migration zone (Fig. 1c). The migration capacity was reduced to 75% at 20 pM (Fig. 1b, left graph), but IFNβ also affected the cell viability endpoint at this concentration (reduction of the cell number by ~16%, *p* value <0.01). As no dead or dying cells were observed at any time point, the apparent ‘reduction of viability’ was most likely due to the known cytostatic effect of the cytokine (Bekisz et al. 2010).

For interpretation of the data from the MINC assay used here, it is important background information that NCC still proliferate during the assay. Thus, it is possible that drugs that inhibit proliferation, but not migration, show a false positive effect in the assay. Indeed, clear cell cycle inhibitors, like the DNA synthesis inhibitor aphidicolin, have been found to reduce the number of cells found in the migration area at the end of the experiment (Nyffeler et al. 2016). To exclude any effect on proliferation, we used the mitosis inhibitor cytosine arabinoside (AraC, 10 µM) as cell medium additive. Under such test conditions, any proliferation was blocked (Fig. 1d), and the specific effect of cytostatic drugs on migration can be quantified without interference of cell cycle effects. When the assay was repeated in the presence of AraC, IFNβ did not affect the viability endpoint at all, but it still triggered a significant inhibition of migration at 25 pM, and the threshold of 25% reduction (Zimmer et al. 2012, 2014; Nyffeler et al. 2016) was reached at 50 pM (Fig. 1b, right graph). Thus, data from different test formats corroborated the earlier finding that pM concentrations of IFNβ reduced the migration of NCC, independent of any potential effects of the drug on NCC proliferation.

**Fig. 2:**
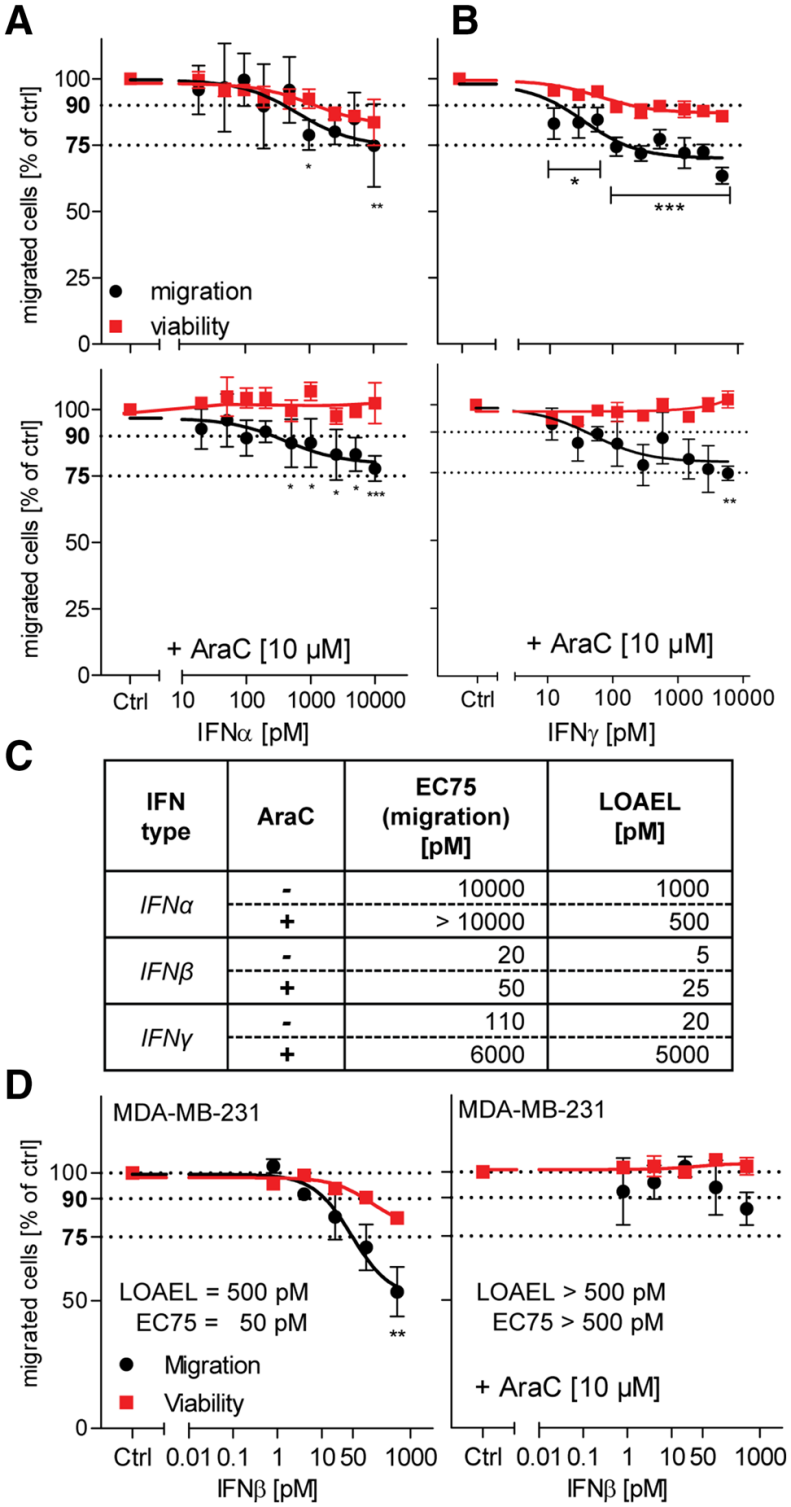
Specificity of IFNβ effects on NCC. NCC were treated for 48 h with interferons, while they were allowed to migrate. Then, the viability and the inhibition of cell migration were measured. All assays were performed either with or without cytosine arabinoside (AraC, 10 µM) as culture medium supplement. **a, b** Testing of interferon-α (IFNα) and interferon-γ **(**IFNγ). Data are from three independent experiments. *Error bars* indicate standard deviations (SD). Statistics was performed for each endpoint by ANOVA, followed by Dunnet’s post-hoc test (*p ≤ 0.05, **p ≤ 0.01, ***p ≤ 0.001). **c** EC_75_ and the lowest observed adverse effect level (LOAEL) were compiled for each scenario. The LOAEL was defined as the lowest concentration triggering a significant reduction of cell migration (*p* ≤ 0.05).** d** Human breast cancer cells MDA-MD-231 were allowed to migrate for 48 h, before viability and the number of migrated cells were quantified. Cells were treated with the indicated concentrations of IFNβ for the total migration period, either with or without cytosine arabinoside (AraC, 10 µM) as culture medium supplement

**Fig. 3:**
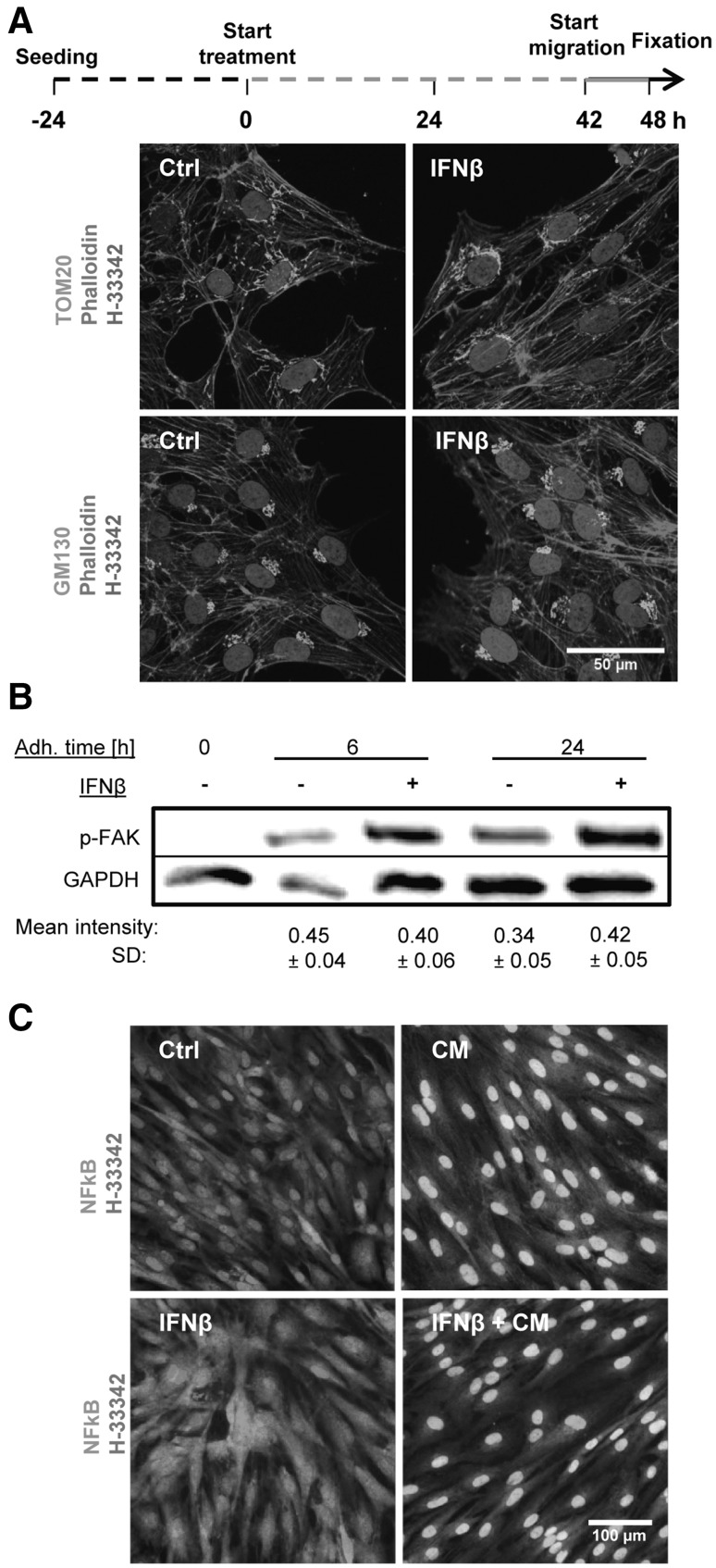
Maintenance of basic NCC functions and morphology in the presence of IFNβ. **a** NCC were treated with IFNβ (500 pM) or solvent (Ctrl) for 48 h, allowed to migrate in the last 6 h of the treatment period, and finally fixed for immunofluorescence staining. The microfilament cytoskeleton was visualized by phalloidin; antibodies to TOM20 were used to visualize mitochondria and anti-GM130 for the Golgi apparatus. **b** NCC were seeded for 0, 6, and 24 h (adhesion time) in culture medium supplemented with IFNβ (500 pM) or solvent. Then, cells were lysed and the amount of phosphorylated FAK was measured by Western blot analysis. The mean intensity of each band normalized to the respective loading control (GAPDH) ± SD is reported below each condition (*n* = 3). In the control (0 h = non-adherent cells), no band was detected. No significant change was observed between control and treatments at 6 and 24 h. **c** NCC were exposed to a cytokine mix (CM, 10 ng/ml TNFα, and 10 ng/ml IL1β), IFNβ (500 pM) or a combination of both for 1 h. Cells were then fixed and stained for nuclear factor kB (NFkB, *green*). Representative pictures for each condition are shown, with nuclei counterstained with H-33342 (*red*). Nuclei with translocated NFkB appear *yellow*, instead of *red* (non-translocated NFkB). (Color figure online)

To investigate whether the effects observed were specific for IFNβ within the interferon family, related cytokines were also tested: IFNα and IFNγ. IFNα binds to the same receptor as IFNβ, but with lower affinity. Accordingly, it affected migration only at much higher (two orders of magnitude) concentrations (Fig. 2a). In contrast to this, the type II interferon IFNγ affected viability and the migration endpoint at low pM concentrations, similar to IFNβ (Fig. 2b). To unambiguously separate the effects on proliferation from those on migration, the interferons were tested under non-proliferating assay conditions (Fig. 2a, b), and both the 75% effective concentration (EC75) as well as the lowest effective concentration (LOAEL) were determined for the migration endpoint and compared to IFNβ (Fig. 3c). These experiments showed that IFNα inhibits migration of NCC, but that it was about two orders of magnitude less potent. The data also showed that IFNγ mainly affected the NCC proliferation at low pM concentration, while specific effects of migration were only observed in the nM range. Thus, IFNβ is the only tested interferon that affected the migration of NCC at clinically relevant low pM concentrations.

As migration is a fundamental cell biological process, it may be assumed that the capacity of IFNβ to disturb cell movement may apply to many other cell types. However, it was shown earlier that many toxicants affect NCC migration without affecting, for example, tumor cells or other neural precursors (Zimmer et al. 2012). To study such specificity for IFNβ, we used the human breast cancer cell line MDA-MD-231, which is highly metastatic, displays a mesenchymal/fibroblastoid phenotype (Prat et al. 2010), and thus migrates in a somewhat similar way as NCC. Under proliferating conditions, IFNβ showed potent effects (low pM range) on both the migration and the viability endpoints (Fig. 2d, left graph). Under test conditions that avoided any drug effects on proliferation, no inhibition of migration by IFNβ was detected (Fig. 2d, right graph). Thus, the effect of IFNβ on the migration of cells did not apply to any cell type, but may be rather specific for NCC.

### Maintenance of basic functions and cell morphology after exposure to IFNβ

One straightforward explanation for the relatively specific effects of IFNβ on NCC migration may be a change of the cell differentiation state by the cytokine. For instance, a mesenchymal-to-epithelial (MTE) transition or a differentiation to a neural or other final cell type may reduce spontaneous migration of the cells. Therefore, various cell features were studied. We examined several typical cell type-specific mRNA markers (e.g., MSX1, SOX9, SNAIL2, NRP1, and PAX3), and none of them changed their expression level upon exposure to IFNβ (suppl. Fig. S4). Moreover, the characteristic cell shape was maintained. To assess the latter finding in more detail, some molecular markers of cell structure were studied. NCC were treated with a maximal concentration of IFNβ (500 pM) for 48 h. Then, the F-actin microfilaments were visualized with phalloidin. Mitochondrial structures were stained with an antibody to TOM20, and the Golgi apparatus was stained for the GM130 protein. Multiple images (>15) were recorded for each conditions, and two observes blinded to the treatment condition examined the cell features. No differences were observed that could be related to the treatment with IFNβ (Fig. 3a).

In a next step, various functions associated with cell adhesion were studied. The expression of 20 integrins and cadherins was studied, and none of them changed >30% in the presence of IFNβ (suppl. Fig. S5). In a more functional approach, we assessed the phosphorylation (and activation) level of the focal adhesion kinase (FAK). Protein samples were prepared from NCC after 0, 6, or 24 h of adhesion, either with or without IFNβ (500 pM). Adhesion of the cells led to the activation of FAK, as expected. This process was not significantly affected by exposure to IFNβ (Fig. 3b).

In a third approach, we examined whether IFNβ may affect the response of cells to external stimuli, such as mitogenic or inflammatory cytokines. NCC were incubated without the growth factors EGF/FGF overnight, and then exposed to these cytokines in the presence of absence of IFNβ. IFNβ alone neither triggered the MAP kinase pathways (as measured by phosphorylation of Erk) nor the Akt pathway (as measured by phosporylation of Akt). EGF/FGF clearly triggered these pathways and this effect was not affected by IFNβ (suppl. Fig. S6). Alternatively, NCC were exposed to inflammatory cytokines (CM: TNFα and IL1β) to probe the response of the NFkB pathway. The CM triggered translocation of the transcription factor NFkB from the cytosol to the nuclei (Fig. 3c, upper panels) in 91 ± 7% of all cells. IFNβ itself did not trigger NFkB translocation. Neither did it affect the signaling of the CM. (Fig. 3c, lower right panel). Thus, no obvious structural features and none of the tested signaling functions were impaired by exposure to IFNβ.

### Attenuation of NCC migration speed and chemotactic behaviour by IFNβ

**Fig. 4:**
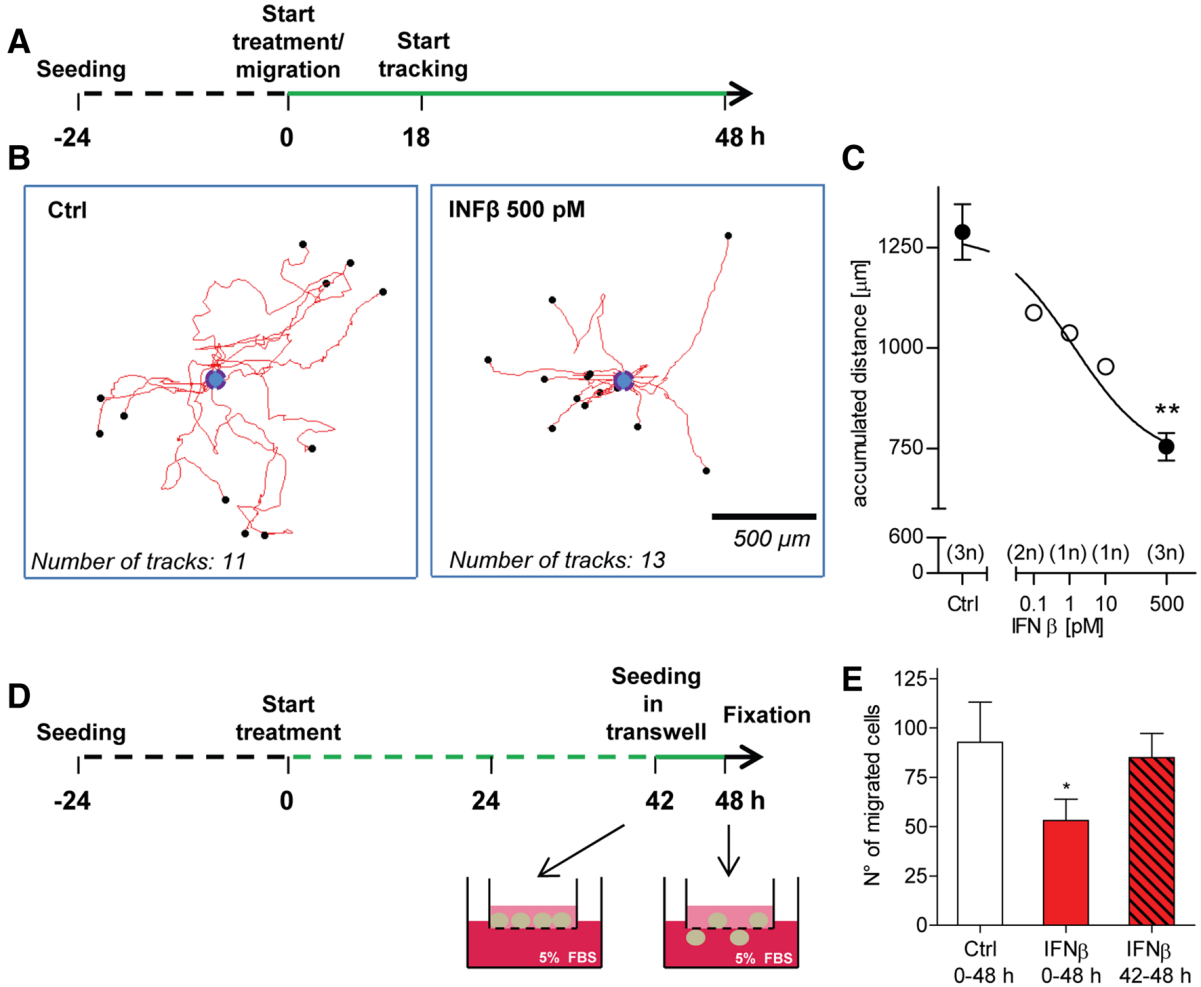
Confirmation of impaired NCC migration in the presence of IFNβ in secondary functional assays. **a** NCC were allowed to migrate for 48 h, and they were exposed to IFNβ at the indicated concentrations for the entire migration period. Phase contrast images were taken every 15 min during the last 30 h of migration for cell tracking. **b** Representative migration tracks for the control and 500 pM IFNβ-treated cells are shown. Tracks are normalized to the same starting point (*blue circle*); *x* and *y* dimensions are scaled similarly (see *scale bar*). **c** Averaged accumulated distance covered by the cells was then calculated for each test concentration. At least 10 cells were followed for each condition (= 1 technical replicate). The number of independent replicates for each condition is reported in the graph. *Error bars* indicate standard deviations (SD). Statistics is based on *t* test analysis between control and maximal IFNβ concentration samples (**p ≤ 0.01); an ANOVA across all data, followed by Dunnet’s post-hoc test yielded the same significance level for 500 pM IFNβ. **d** NCC pre-treated for 42 h with IFNβ or solvent were seeded into transwells. Then, the cells were induced to migrate through the transwell porous membrane by addition of 5% FBS into the lower chamber of the transwell, in the presence of IFNβ (500 pM) or respective control. After additional 6 h in the presence or absence of IFNβ, migrated cells were stained with crystal violet. Four fields per replicate were imaged, and the cells were counted. **e** Quantification of the number of migrated cells for the different conditions: the total IFNβ exposure period is indicated; data are from four independent experiments. *Error bars* indicate standard deviations (SD). *p ≤ 0.05. (Color figure online)

To confirm the impairment of NCC migration by IFNβ, and to further exclude any artefacts due to drug effects on cell proliferation or differentiation, two other functional assays were used. The first approach made use of time lapse imaging and cell tracking to study effects on cell speed. Migrating cells were incubated with IFNβ on a microscope stage in a temperature-controlled incubation system (Fig. 4a). This setup allowed a continuous recording of phase contrast images for 30 h. The image stacks were then used to track the migrating cells (≥10 cells per well) (Fig. 4b). Analysis of the tracks revealed a concentration-dependent reduction of the accumulated distance travelled by IFNβ-treated NCC, compared to control cells (Fig. 4c). Thus, this method confirmed on the single cell level that IFNβ reduced the migration speed.

The second approach made use of a transwell set-up. In this system, cells can be seeded onto a porous membrane in the upper compartment. When a chemoattractant (here FBS was used) is added to the bottom part, cells are triggered to migrate through the membrane. NCC were seeded on the transwell membrane directly or after a 42-h pre-treatment with IFNβ. At the end of a 6-h migration period, cells that had translocated through the membrane were counted (Fig. 4d). NCC treated with IFNβ for the period from 0 to 48 h showed a significant reduction of their capacity to migrate through the membrane. NCC treated only during the last 6 h of the migration period (42–48 h) were not impaired (Fig. 4e). This set of data suggests that (1) the migration inhibition by IFNβ can also be measured in a different (here 3-dimensional) test setup; and (2) that cells must be exposed for >6 h to IFNβ for the inhibition of migration to become apparent.

### Transcriptome changes and alterations of superordinate biological processes induced by IFNβ in NCC

**Fig. 5:**
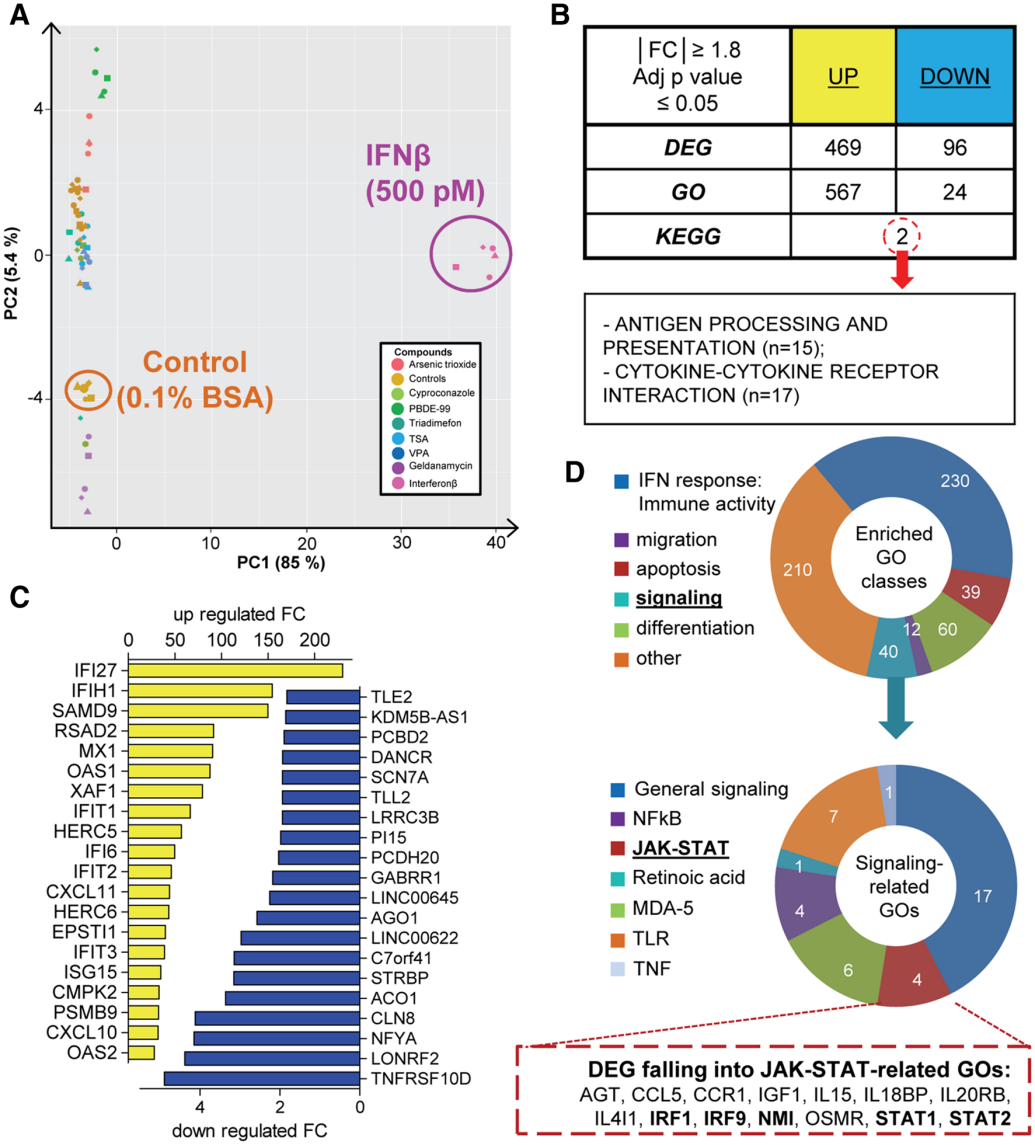
Transcriptome changes triggered by IFNβ in NCC. Sampling for microarray analysis was performed in NCC after 48-h exposure to non-cytotoxic, but migration-inhibiting, concentrations of eight test battery hits, as identified in Zimmer et al. (2014). Data are from five independent experiments (= data points of one colour, but different *shapes*). **a** Principal component analysis (PCA) was performed, and a 2D plot was generated to display the transcriptome data structure across compounds and experimental replicates. The positions of IFNβ-exposed samples, and the respective control, are circled. On the axes, the first two principal components are plotted, and the percentage of covered variances is reported. **b** Number of differentially up-regulated (*UP*) or down-regulated (*DOWN*) genes (DEG, IFNβ vs control) and the corresponding biological processes (over-represented GO classes) was identified. Over-represented KEGG pathways were searched amongst all DEG (*UP* and *DOWN*), and the only two significant pathways are indicated. **c** Identified DEG were sorted according to their *p* value. The top 20 up- (*yellow*) and down- (*blue*) regulated genes are shown as bar graphs indicating the fold change (FC). Large regulation factors were observed especially for genes that showed very low expression in untreated cells (as is common for transcriptome analysis of inflammatory situations). The effect is unlikely to be due to baseline variations, as the sorting was done according to the *p* value for the regulation. **d** *Ring diagrams* show the relative distribution of 6 superordinate biological processes (IFN response, migration/chemotaxis, apoptosis, signaling, differentiation, and other) amongst the over-represented GO classes (*upper ring*) and the number of the different signaling-related over-represented GO classes (*lower ring*). Genes with a central role in the JAK-STAT pathway are depicted in bold. (Color figure online)

To obtain more data on the type of effects IFNβ has on NCC, cells were exposed for 48 h to the cytokine (500 pM). Then, mRNA was prepared from five different cell lots and used for gene expression analysis by Affymetrix microarray technology. The data were compared to those obtained for seven toxicants and pharmaceuticals (arsenic trioxide, cyproconazole, PBDE-99, triadimefon, TSA, VPA, and geldanamycin) known to inhibit the migration of NCC (Zimmer et al. 2014; Pallocca et al. 2016). Each of the compounds was used at its respective highest non-cytotoxic concentration, and the data structure was visualized by a principal component analysis (Fig. 5a). Plotting of the first two principal components showed a strong separation between IFNβ-treated samples and all other samples along the first principal component axis. This was due to the much stronger gene regulation response triggered by IFNβ, compared to small molecular weight toxicants.

The gene expression data were then used to determine the differentially expressed genes (DEG). The DEG for each toxicant were defined here as the group of microarray probe sets (PS), which differed significantly from negative controls (FDR adjusted *p* value ≤0.05), and showed expression level changes (fold change (FC)) of ≥1.8 or ≤0.55 (Falsig et al. 2004; Pallocca et al. 2016). IFNβ triggered a much stronger up-regulation than down-regulation response, altering altogether 565 PS (Fig. 5b). To get more insight into the deregulated genes, the DEG were sorted according to their *p* values and the top 20 up- and down-regulated genes were displayed. Among the most strongly up-regulated genes, there were many IFN-induced proteins (IFI), which have mostly roles in the inhibition of viral replication. Other genes were the 2′–5′ oligoadenylate synthetase family (OAS1/2), which activates the latent RNase L, and induces viral RNA degradation; another typical biomarker of IFN anti-viral responses was the MX dynamin like GTPase (MX1). In addition, we found a strong up-regulation of the chemokine response (CXCL10 and 11). The group of strongly down-regulated genes was more heterogeneous. It comprised, for instance, the TNF receptor superfamily member TNFRSD10D, argonaute 1 (AGO1, the catalytic component of the RISC complex involved in the RNA interference process), and the zinc-dependent metalloprotease tolloid-like (TLL2) (Fig. 5c).

As the analysis of individual genes did not directly indicate which migration-related pathways may be impaired, we investigated whether biologically linked groups of genes were co-ordinately regulated. More than 500 gene ontology (GO) terms were enriched (adjusted *p* value ≤0.05) amongst the up-regulated genes, while only 24 terms were over-represented amongst the down-regulated genes. To get a better idea on co-ordinately affected pathways, we also identified over-represented KEGG pathways amongst the sum of DEG. The two pathways found by this approach were “antigen processing and presentation” (15 genes represented) and the “cytokine-cytokine receptor interaction” pathway (17 genes represented). This was not surprising, given a main role of interferons in these processes (Fig. 5b), but it did not offer an evident explanation, why migration was impaired.

To get an overview of the over-represented GO groups, we assigned them to six superordinate cell biological processes (Waldmann et al. 2014): IFN responses, migration, apoptosis, signaling, differentiation, and others. As expected, most of the enriched GO classes belonged to the “IFN response group” (230 GO term groups). We focused on the signaling-related over-represented GO terms (40 groups) to get some hints regarding the mode of action of IFNβ on the migration capacity of NCC. Several GO in this group related to innate immunity and inflammatory signaling. Amongst the more general signal transduction pathways, only the JAK-STAT pathway was clearly over-represented (4 GO groups) with several key players of this pathway being up-regulated (Fig. 5d). For this reason, we decided to examine the role of JAK-STAT signaling on NCC migration more directly.

### Correlation of IFNβ-induced inhibition of NCC migration and JAK-STAT pathway activation

**Fig. 6:**
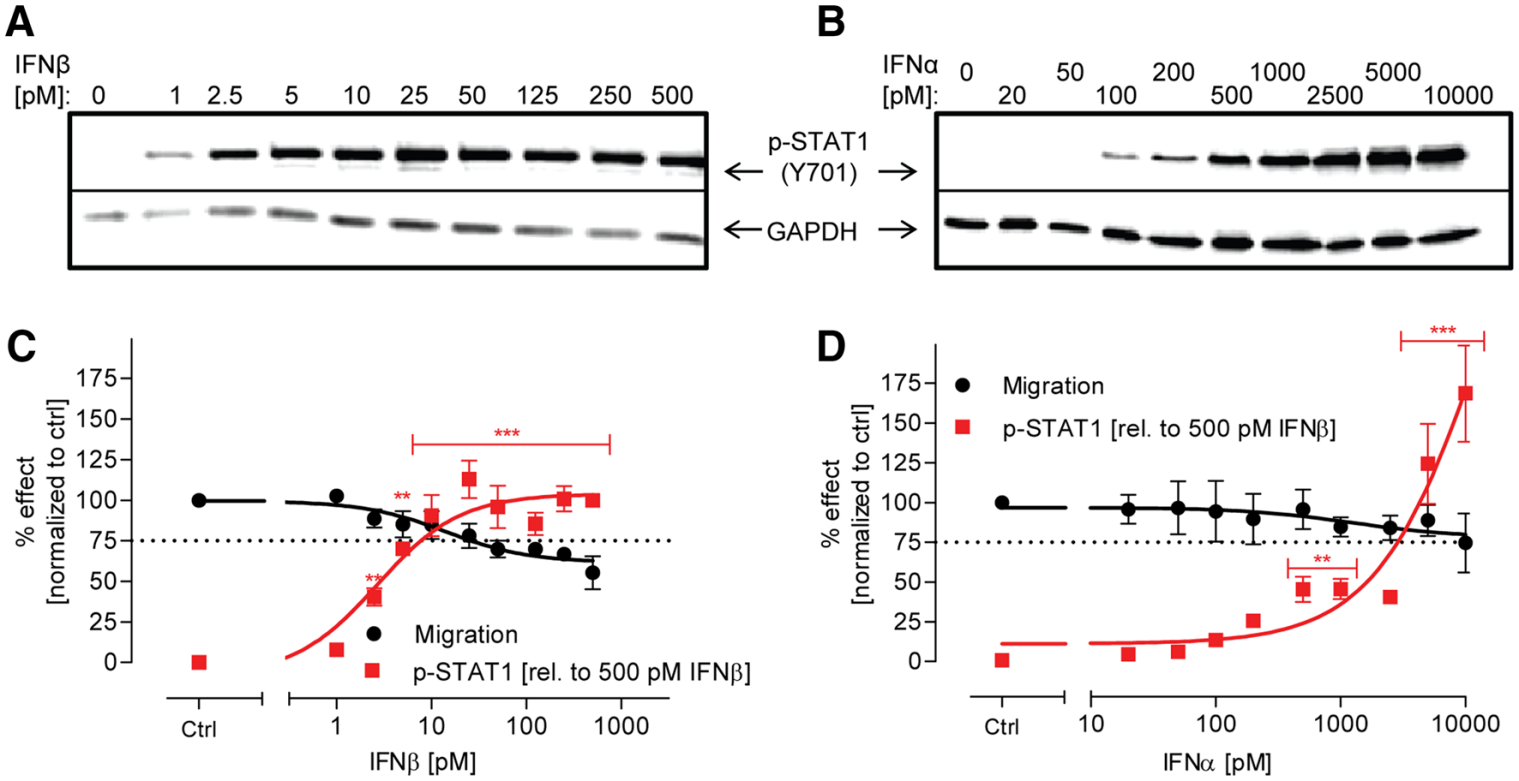
Correlation between JAK-STAT pathway activation and inhibition of NCC migration upon treatment with class I IFN. **a, b** NCC were exposed to the indicated concentrations of IFNβ and IFNα for 1 h. Then, cells were harvested and protein samples were prepared. The amount of phosphorylated STAT1 (p-Tyr701) was measured by western blot analysis (representative blots are shown). **c, d** Band intensity was quantified and normalized to the respective GAPDH antibody band. For better comparison, the migration inhibition data from Fig. 1b are shown in *black* in the same graph. Data are means from three independent experiments. *Error bars* indicate standard deviations (SD). Statistical analysis was based on ANOVA, followed by Dunnet’s post-hoc test (*p ≤ 0.05, **p ≤ 0.01, ***p ≤ 0.001)

As readout for JAK-STAT activation, we chose to analyze the phosphorylation level of one of the members of the STAT family, STAT1. This step is necessary for the heterodimerization of STAT proteins, which together with IRF9 form, and then the transcription factor IFN-stimulated gene factor 3 (ISGF3), and promotes IFN-induced genes transcription. Western blot analysis showed an increase of p-STAT1 in NCC after stimulation with both IFN class I interferons, with IFNα being about 100-fold less potent than IFNβ (Fig. 6a, b). The extent of STAT1 phosphorylation was quantified, and it correlated with the extent to which migration was inhibited. This data set showed clearly that p-STAT1 levels correlated with the inhibition of migration in the case of IFNβ treatment (Fig. 6c). To reach 50% of STAT1 phosphorylation, NCC needed to be exposed to 2 nM of IFNα (vs 5 pM IFNβ), but also for IFNα, the increase of pSTAT1 correlated with inhibition of migration (Fig. 6d). To evaluate a specific role of JAK-STAT signaling, we also examined phosphorylation of extracellular signal regulated kinases (ERK1/2), of protein kinase B (AKT), and of glycogen synthase kinase-3β (GSK3β), but none of them was changed by exposure to IFNβ (suppl. Fig. S6).

**Fig. 7:**
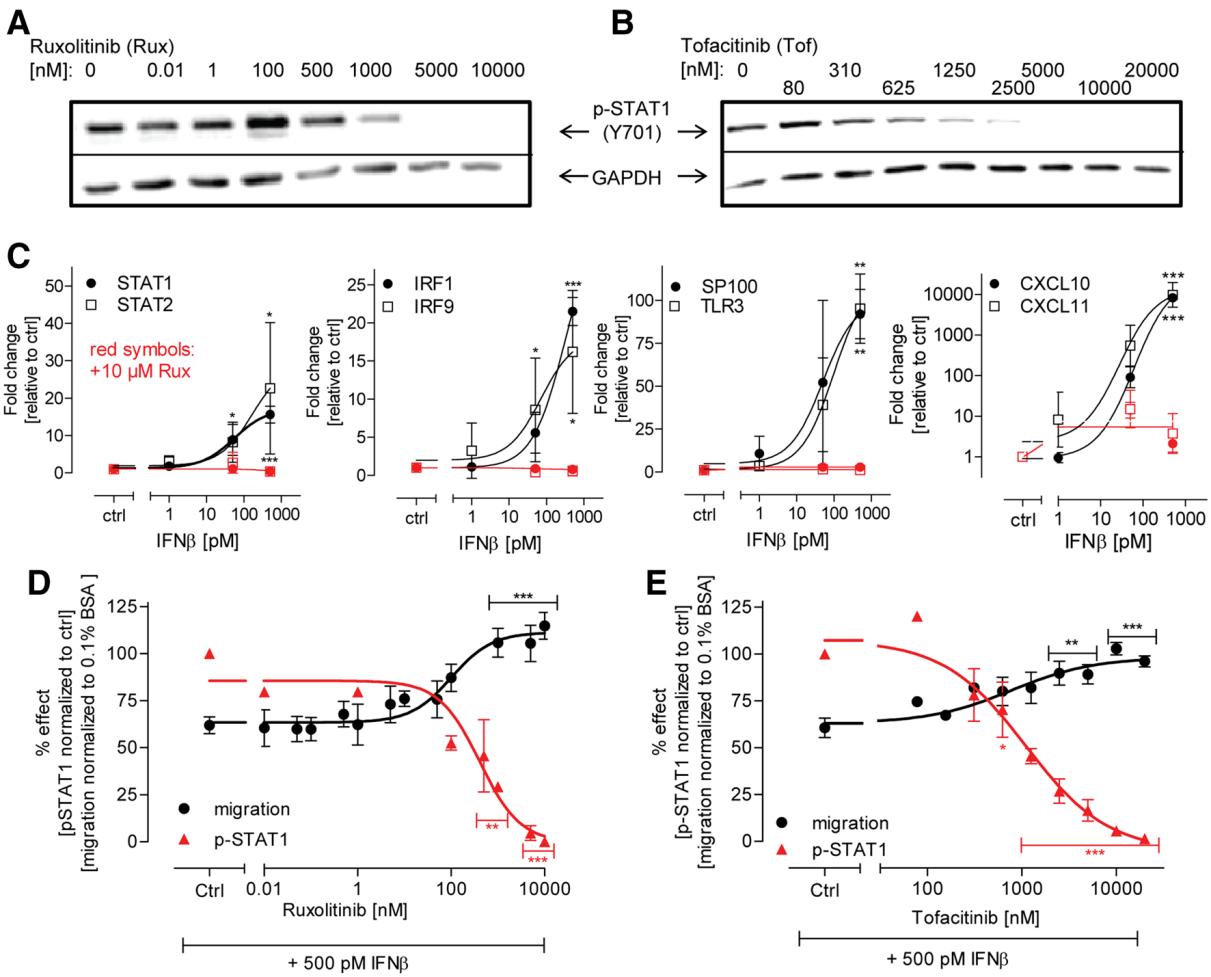
Abolishment of the effect of IFNβ on NCC migration by inhibition of the JAK-STAT pathway. **a, b** NCC were pre-treated for 0.5 h with two different JAK inhibitors (ruxolitinib and tofacitinib) at the indicated concentrations. Then, the cells were further treated for 1 h with the inhibitors in cell culture medium supplemented with 500 pM IFNβ. Finally, cells were harvested and the amount of phosphorylated STAT1 (p-STAT1) was measured by western blot analysis (representative *blots* are shown). **c** NCC were exposed to IFNβ for 48 h at the indicated concentrations, either with or without ruxolitinib (Rux, 10 µM). Then, cells were harvested, and total RNA was extracted and retro-transcribed. Effects on selected mRNAs were evaluated by qPCR. Expression levels were normalized against the housekeeping gene, GAPDH and are expressed relative to control levels (untreated cells). The mRNA expression in the presence of Rux is shown in *red*. Note that the *red symbols* are often overlapping, due to complete inhibition down to control levels. **d, e** Band intensities were quantified for p-STAT1 (normalized to GAPDH). In a parallel set of experiments, migration (after 48 h) was evaluated in the presence of IFNβ (500 pM) plus ruxolitinib (*left*) or tofacitinib (*right*). Data are from three independent experiments. *Error bars* indicate standard deviations (SD). Statistics was performed by ANOVA, followed by Dunnet’s post-hoc test (*p ≤ 0.05, **p ≤ 0.01, ***p ≤ 0.001). (Color figure online)

To obtain more causal evidence for the role of the JAK-STAT pathway in the inhibition of NCC inhibition, we used two different and specific JAK inhibitors (ruxolitinib and tofacitinib) to block the kinase signaling. Both inhibitors blocked STAT1 phosphorylation concentration-dependently (Fig. 7a, b). Moreover, exposure to ruxolitinib completely abolished the activation of eight different interferon target genes in cells exposed to IFNβ (Fig. 7c). A pharmacological block by any of the two inhibitors of the pathway resulted in the complete prevention of adverse effects of IFNβ. Moreover, the extent of signaling pathway inhibition correlated significantly (*p* < 0.01) with the rate of reversal of the intereferon-beta effect (Fig. 7d, e).

### Requirement for continued JAK-STAT signaling for impairment of NCC migration

Having established that the adverse effects of IFNβ on the migration of NCC are signaled via the JAK-STAT pathway, we were interested in the role of short vs continued exposure to interferons. To address this question, we exploited the rescuing effect of the JAK inihibitor ruxolitinib to terminate the IFNβ signaling at defined time points.

**Fig. 8:**
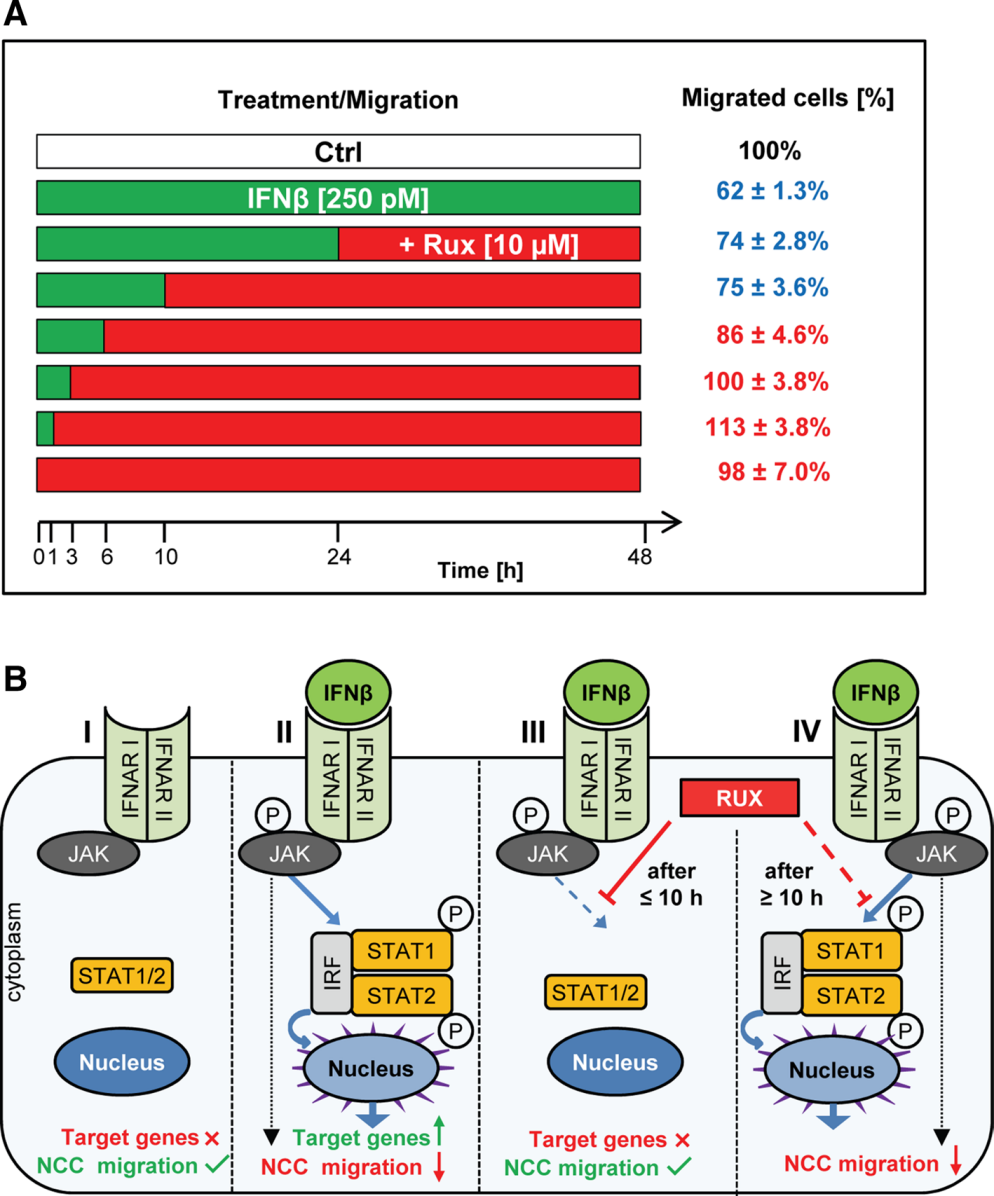
Requirement for continued JAK-STAT signaling to NCC migration impairment. **a** NCC were allowed to migrate for 48 h. During this time, they were exposed to IFNβ while ruxolitinib (10 µM) was added to the culture medium at different time points after the start of the migration. The percentage of migrated cells was quantified for all conditions after 48 h. Migration was inhibited ≥25% in all conditions denoted in *blue*. Ruxolitinib without IFNβ had no effect on migration. **b** Graphical representation of the effects of IFNβ and JAK-STAT pathway-inhibitors on the transcription of target genes and on the functionality of NCC. NCC migration was unimpaired in the absence of IFNβ or when IFNβ was present together with ruxolitinib. NCC migration was impaired when IFNβ was present alone, or when ruxolitinib was added ≥10 h after IFNβ. (Color figure online)

The standard migration assay, as described in 3.1, was performed in the presence of IFNβ (or respective control). Ruxolitinib was added to the cells immediately or at various time points after exposure to interferon. The inhibition of migration was then evaluated at 48 h for various experimental conditions (Fig. 8a). The results from these experiments showed that the inhibition of migration (by IFNβ) was rescued when the inhibitor ruxolitinib was added not later than 6 h after the beginning of IFNβ treatment. When ruxolitinib was added after 10 h (or later), inhibition of migration (by at least 25%) occurred (Fig. 8b). Thus, inhibition of migration by IFNβ required receptor signaling for at least 6–10 h, while activation of the JAK-STAT pathway for up to 6 h hardly affected the migration capacity of NCC (Fig. 8b).

This finding was corroborated when standard migration assays were performed and IFNβ was washed out after 3 h. Under these conditions, no inhibition of cell migration was observed (data not shown).

## Discussion

In the present study, a potential developmental toxicity hazard of IFNβ with regard to NCC disturbance was confirmed. Migration of NCC was affected at low pM concentrations by IFNβ, while IFNα was substantially (two orders of magnitude) less potent, and IFNγ did not affect migration in the pM range. The effect on migration clearly differed from the cytostatic activity of the cytokines, as (1) IFNβ and IFNα had similar cytostatic potency on the test system, and (2) as cytostatic and migration-inhibiting properties could be experimentally separated. Moreover, the specific effect of IFNβ on migration was confirmed in assays with different protocols and endpoints. A closer cell biological characterization showed that the inhibition of migration correlated well with a prolonged activation of the JAK-STAT signaling pathway in various sets of experiments. Thus, prolonged activation of STAT1 by IFNβ in NCC provides a biochemical marker of potentially adverse effects of IFNβ on fetal development during pharmacological use.

As IFNα and IFNβ signal through the same receptor complex, the largely different potencies observed here may appear surprising. However, such observations are explained by the higher affinity of IFNβ (compared to IFNα) for the shared receptor subunits. A more pronounced effect of IFNβ has also been observed in other test systems. For instance, only IFNβ induced JAK1 activation in human myocardial fibroblasts, and this was associated with a 120-fold higher potency to trigger an anti-viral response (vs IFNα) (Heim et al. 1996; Grumbach et al. 1999); also in human vascular endothelial cells, a 2–3 log difference was observed for IFNβ vs IFNα in functional assays (da Silva et al. 2002), and IFNβ was 100-fold more potent than IFNα with respect to the inhibition of differentiation of human monocytes into osteoclasts (Coelho et al. 2005). Notably, the difference in affinity is only relevant for the biological effect in cells that express low numbers of receptors; in cells with a high receptor reserve, functional effects are observed for both type I interferons within the same concentration range (van Boxel-Dezaire et al. 2006; Moraga et al. 2009; Schreiber and Piehler 2015). As high and low receptor numbers are not an absolute measure, but relate to the percentage of receptors required to trigger a full biological response in a given cell type, the potency difference of IFNα and IFNβ may differ for different responses within one given cell (Zula et al. 2011; Piehler et al. 2012). From these considerations, widely accepted in the interferon literature, we conclude that NCC express low numbers of the receptor subunits IFNAR1 and IFNAR2 on their surface. Moreover, we may conclude that a higher occupancy is required to affect migration than to trigger a cytostatic effect.

To our knowledge, effects of IFNβ on the migration of neural cells or their precursors have not been reported yet. However, the migration and invasion properties of glioma cells are affected by interferon-regulated genes (Yu et al. 2011; Tarassishin and Lee 2013) and neuronal survival seems to require a basic level of interferon signaling (Ejlerskov et al. 2015). Moreover, interference of IFNβ with migration has been shown especially in the leukocyte lineage of cells (Lou et al. 1999; Staun-Ram and Miller 2011; Floris et al. 2002) and for various tumor cells (Booy et al. 2015; Rossi et al. 2015). Many of these studies suggest that cell migration may be affected by interferons through a reduced secretion of matrix metalloproteases (Stuve et al. 1997; Yen et al. 2010). This mechanism is unlikely to play a role in the experimental systems used in our study, as the penetration of extracellular matrix is not required for two-dimensional NCC migration. As IFNβ is extremely pleiotropic in its activities, great care is required to distinguish effects that are correlated with reduced migration and effects that causally lead to reduced migration.

In the present study, we found that JAK-STAT signaling is a causal factor involved in reduced migration. In parallel, we invested major efforts to avoid erroneous conclusions, based on secondary effects triggered by IFNβ. These experiments followed two main lines. First, we made sure that migration was specifically affected, and that test results were not an indirect consequence of the known cytostatic effects of interferons (Bekisz et al. 2010). These precautions are particularly important for slowly migrating cells (as NCC), while such controls are less common in the field of leukocyte migration. One taken approach was to run the assay under conditions that did not allow any proliferation (presence of a mitosis inhibitor). IFNβ showed its effect on migration also in this setup. The second approach was to use an assay with a faster readout (transwell assay), and then, going one step further, we followed individual cell migration directly by tracking. In addition, these experiments confirmed the inhibition of migration in a low pM range of IFNβ.

Second, we provided evidence that IFNβ treatment did not obviously alter the cell differentiation state. In theory, extracellular signals may lead to the differentiation of NCC to neurons or other progeny that are less prone to migration. This is unlikely to happen in our test system, as several differentiation markers remained unchanged, as a characterization on the organellar level did not reveal differences after treatment with IFNβ, and as key molecules relevant to adhesion and migration, like focal adhesion kinase or expressed integrins, remained unaffected. The stability of the overall NCC phenotype is remarkable, especially in the light of the transcriptome analysis, which showed that a typical (anti-viral and immunomodulatory) interferon response was triggered on the level of gene expression. Most conspicuous amongst these regulated transcripts were several chemokines and chemokine receptors (CCL5, CXCL10, CXCL11, and CCR1), but the changes were observed too late to be relevant for the effects on migration observed here. Moreover, the migration of NCC was not affected in our assay by chemokine receptor antagonists (not shown).

Several sets of data presented here suggest that the extent of activation of the JAK-STAT pathway correlates with inhibited migration by IFNβ. Moreover, we also explored the temporal relationship of this pathway activation with NCC motility. It is known that cytokines, like interferons, may either trigger a hit-and-run signaling response (Brask et al. 2004), i.e., short formation of the receptor-ligand complex has long-acting or irreversible effects, or they may lead to an acute cellular change reversible after termination of cytokine–receptor interaction. In other cases of cytokine action, prolonged receptor signaling is required to trigger downstream cellular processes, and once triggered the cellular alterations may remain stable. Here, we found an example of the latter response pattern: activation of the JAK-STAT pathway for less than 6 h had no significant effect on cell migration; activation for more than 10 h was sufficient to trigger the full response, also when the kinase pathway was then blocked by specific inhibitors. This suggests that short peak levels of the cytokine would not affect NCC migration in a fetus, but if such levels are maintained in chronic infection, or after transfer of pharmacologically applied IFNβ across a compromised placental barrier, adverse developmental effects may follow.

In this context, it is important that interferons show a high degree of species-specificity. The evaluation of the effects of human IFNβ is, therefore, not possible in rodent or rabbit models, and only some monkeys (e.g., rhesus monkeys) show pharmacodynamic responses to the cytokine. Concerning the pharmacokinetic behaviour, the most relevant data come from clinical trials. IFNβ is normally given to MS patients intramuscularly at a dose of 6 million international unites (MIU) (Barbero et al. 2004). In controlled pharmacokinetic studies, 12 MIU IFNβ given intramuscularly to healthy patients led to a C_max_ of 44 IU/ml, corresponding to ~9 pM (Alam et al. 1997). A rough linear approximation would suggest that the clinical dose used in MS would lead to peak plasma levels of 4–5 pM, i.e., roughly within the range of the concentrations found to affect NCC here. In a previous study (Zimmer et al. 2014), we calculated the dose to result in developmental toxicity to monkeys (FDA 1999) to correspond to plasma levels of 540 pM. This concentration is higher than the concentrations found here to affect human NCC, but the data basis is relatively limited and the concentration estimate requires several assumptions for the PBPK modelling (Zimmer et al. 2014). Unfortunately, retrospective clinical studies do not provide a clearer set of information. The safety of IFNβ in pregnancy has been observed on several occasions, but with contrasting results (Sandberg-Wollheim et al. 2005). To date, women with MS are advised to interrupt the exposure to the drug for precautionary reasons (Lu et al. 2012; Pozzilli and Pugliatti 2015), but information on no-effect levels or critical windows of susceptibility are not available.

In the absence of clear clinical data, and in the pertinent case of highly species-specific effects of the drug, our human cell-based testing approach allowed important insights into potential toxicological hazard, and into the concentration range at which this may be expected. In this context, it is important that the phenotypic adverse effect (inhibited migration) was consistent with a plausible biochemical mechanism of IFNβ signaling (JAK kinase activation), that exact information on the relevant concentration range was obtained, and that several tests and endpoints confirmed the type of potential hazard identified in initial studies. On the basis of this example study, we suggest that a combination of cellular tests based on relevant human cell types and engineered tissue structures (e.g., placental barrier) be used more frequently to obtain information on toxicological properties of human-specific drugs.

## Electronic supplementary material

Below is the link to the electronic supplementary material.

Supplementary material 1 (XLSX 577 KB)

Supplementary material 2 (PDF 345 KB)

## Abbreviations

AKT: Protein kinase B
AraC: Cytosine arabinoside
CM: Cytokine mix
TNFα: Tumor necrosis factorα
DEG: Differentially expressed genes
EdU: 5-Ethynyl-deoxyuridine
ERK: Extracellular signal regulated kinase
ESNATS: Embryonic stem cell-based novel alternative testing strategies
FAK: Focal adhesion kinase
FC: Fold change
FDA: Food and drugs administration
FDR: False discovery rate
GFP: Green fluorescent protein
GO: Gene ontology
EC: Effective concentration
GSK3β: Glycogen synthase kinase 3β
hESC: Human embryonic stem cell
IFI: Interferon inducible protein
IFN: Interferon
IFNAR: Interferon alpha and beta receptor
IL-1β: Interleukin 1β
IRF: Interferon regulatory factor
ISG: Interferon stimulated gene
ISGF: Interferon stimulated gene factor
JAK: Janus kinase
LOAEL: Lowest observed adverse effect level
MINC: Migration inhibition of neural crest
MIU: Million international units
MS: Multiple sclerosis
MTE: Mesenchymal-to-epithelial
NC: Neural crest
NCC: Neural crest cell
PCA: Principal component analysis
PS: Probe set
STAT: Signal transducer and activator of transcription
TSA: Trichostatin A
VPA: Valproic acid
