# Supplementary material for: Impairment of human neural crest cell migration by prolonged exposure to interferon-beta
Source: Arch Toxicol. 2017 Apr 1;91(10):3385–402. doi: 10.1007/s00204-017-1966-1 (PMC5608792; doi:10.1007/s00204-017-1966-1)
Supplement: Supplementary file 2 — Supplementary material 2 (PDF 345 KB) [file 204_2017_1966_MOESM2_ESM.pdf]

Supplemental Material

Impairment of human neural crest cell migration by prolonged exposure to interferon-beta

Giorgia Pallocca, Johanna Nyffeler, Xenia Dolde, Marianna Grinberg, Gerhard Gstraunthaler, Tanja Waldmann, Jörg Rahnenführer, Agapios Sachinidis and Marcel Leist

Table of contents

- **Supplemental material, Figure S1** STR profiles for H9, H9-Dll1 and reference H9 page 2
- **Supplemental material, Figure S2** Primer list page 3
- **Supplemental material, Figure S3** Differentially expressed genes (DEG) of NCC treated with interferon beta (500 pM) compared to control page 4
- **Supplemental material, Figure S4** mRNA expression level of differentiation markers in presence of increasing IFNβ concentrations page 10
- **Supplemental material, Figure S5** RNA expression level of integrins and cadherins in NCC upon exposure to IFNβ page 11
- **Supplemental material, Figure S6** Phosphorylation level of different pathway-specific kinases in presence of IFNβ page 12
- **Supplemental material, Table 1** Differentially expressed genes (DEG) of NCC treated with interferon beta (500 pM) compared to control (excel document)

Cell type / STR	**H9** (performed on February 2017)	**H9-Dll1** (performed on February 2017)	**H9** (Josephson et al 2006)
Amelogenin	X	X	X
CSF1PO	11	11	11
D5S818	11, 12	11, 12	11, 12
D13S317	9	9	9
D7S820	9, 11	9, 11	9, 11
D16S539	12, 13	12, 13	12, 13
vWA	17	17	17
THO1	9.3	9.3	9.3
TPOX	10, 11	10, 11	10, 11
Penta D	9, 13	9, 13	
D8S1179	8, 14	8, 14	
FGA	26, 28	26, 28	
D3S1358	13, 16	13, 16	
D21S11	30	30	
D18S51	12.3, 13	12.3, 13	
Penta E	11, 14	11, 14	

Supplementary figure S1

Fig S1: STR profiles for H9, H9-Dll1 and reference H9 (Josephson et al 2006)

Gene name	Sense primer sequence	Antisense primer sequence
STAT1	CCTCCTGTCACAGCTGGATGATC	GCCTGATTAAATCTCTGGGCGTT
STAT2	GAAGTCGCGACCAGAGCCAT	CAGCTTCCTGCCAGTTCTGG
IRF1	ACCTCTGCCTTCTTCCCTCTTC	ATCCAGATGAGCCCCGGGATTT
IRF9	GCGACAGCCTGGACAGCAAC	GGCAGCATCCTGGTCCTCCC
SP100	TGACAGGCTGCTCTATGACATTGTA	GTACACCACTCTCTGTACAGGGACC
TLR3	TTTCGAGAGTGCCGTCTATTTG	GGCTAACAGTGCACTTGGTGGT
CXCL10	ACCTCCAGTCTCAGCACCATGAA	ACGTGGACAAAATTGGCTTGCAG
CXCL11	CTTGGCTGTGATATTGTGTGCTACA	GGGATTTAGGCATCGTTGTCCTTTA
GAPDH	ATGGAGAAGGCTGGGGCTCA	AGTGATGGCATGGACTGTGGTCAT
MSX1	ACACAAGACGAACCGTAAGCC	CACATGGGCCGTGTAGAGTC
SOX9	AGGAAGCTCGCGGACCAGTA	CTGCCCGTTCTTCACCGACT
COL2A2	GTCTACCCCAATCCAGCAAA	CTTCAGGGCAGTGTACGTGA
SNAI2	CGCGCTCCTTCCTGGTCAAG	CATTGGGTAGCTGCTGGGCGTGG
HDAC1	CACCCGGAGGAAAGTCTG	GGGCGATAGATTTCCATTTTT
NRP1	GCGGACCCGGAGAAGGGAGA	TCCGGAGCCTGAATCAGCCA
PAX3	GAACACGTTCGACAAAAGCA	GCACACAAGCAAATGGAATG

Supplementary figure S2

Fig S2: Nucleotide base sequences of the primers used for qPCR analysis

Supplementary Table S1		500 pM Interferon beta						
Probe set/ Gene name	Fold change	Adj. p value	Probe set/ Gene name	Fold change	Adj. p value	Probe set/ Gene name	Fold change	Adj. p value
ADAR	1.98	<0.001	C19orf66	10.18	<0.001	CTSZ	1.89	0.005
ADCY7	1.83	<0.001	C1R	2.52	<0.001	CX3CL1	2.04	<0.001
AGT	2.03	0.011	C1S	2.70	0.003	CXCL10	31.95	<0.001
AGTRAP	2.38	<0.001	C1S	3.22	<0.001	CXCL11	44.00	<0.001
AGTRAP	1.85	0.001	C3AR1	1.86	0.007	CXCL11	44.23	<0.001
ANKRD45	1.84	0.014	C5orf56	2.20	0.001	CXCL16	1.95	0.022
APOBEC3B	1.93	0.003	C5orf56	2.49	0.002	CYP4V2	2.42	<0.001
APOBEC3G	9.75	<0.001	C5orf56	5.29	<0.001	DDX58	23.12	<0.001
APOL1	3.68	<0.001	CARD16	11.39	<0.001	DDX58	18.07	<0.001
APOL2	3.34	<0.001	CASP1	8.33	<0.001	DDX58	11.35	<0.001
APOL3	3.61	<0.001	CASP1	11.13	<0.001	DDX60	6.94	<0.001
APOL6	5.05	<0.001	CASP1	7.74	<0.001	DDX60L	4.37	<0.001
APOL6	17.11	<0.001	CASP1	9.45	<0.001	DDX60L	11.66	<0.001
APOL6	7.97	<0.001	CASP1	18.07	<0.001	DHRS2	2.96	<0.001
APOL6	7.04	<0.001	CASP4	2.02	<0.001	DHRS2	6.99	<0.001
ARHGAP15	1.98	0.004	CASP7	2.33	<0.001	DHX58	1.95	0.003
ATP10A	6.23	<0.001	CCL5	2.73	0.002	DIEXF	1.92	0.020
B2M	3.21	<0.001	CCL5	2.05	0.010	DLL1	2.44	0.003
B2M	4.88	<0.001	CCR1	5.45	<0.001	DSP	2.34	<0.001
B2M	5.14	<0.001	CCR1	2.95	0.001	DTX3L	22.31	<0.001
BLZF1	2.17	0.006	CD274	3.24	<0.001	ECM2	2.00	0.006
BLZF1	1.91	0.001	CD55	2.18	0.002	EIF2AK2	3.96	<0.001
BNIP3	2.23	0.001	CEACAM1	3.95	<0.001	EIF2AK2	3.67	<0.001
BNIP3	2.15	<0.001	CFB	8.66	<0.001	EIF2AK2	3.92	<0.001
BST2	23.84	<0.001	CHMP5	1.94	0.001	ELOVL3	1.97	0.002
BTC	2.17	0.007	CLDN1	2.08	<0.001	EPSTI1	39.77	<0.001
BTN3A1	2.07	0.003	CLDN23	3.49	<0.001	EPSTI1	28.55	<0.001
BTN3A1	2.67	0.001	CLEC2B	3.55	<0.001	ERAP1	2.26	<0.001
BTN3A2	4.27	<0.001	CLEC7A	2.18	0.006	ERAP1	3.36	0.009
BTN3A2	3.55	<0.001	CLIC2	2.17	0.006	ERAP1	3.00	<0.001
BTN3A3	3.58	<0.001	CMPK2	32.95	<0.001	ERAP2	5.21	<0.001
BTN3A3	5.74	<0.001	CNP	2.29	<0.001	ERAP2	5.79	<0.001
C19orf66	5.37	<0.001	COL15A1	2.07	0.003	ERAP2	2.19	0.008
C19orf66	3.72	<0.001	CTSS	2.87	0.001	ERG	1.93	0.026
C19orf66	10.18	<0.001	CTSS	2.54	0.001	ETV7	1.87	0.003

Supplementary figure S3 (1)

**Supplementary Table S1**		**500 pM Interferon beta**						
Probe set/ Gene name	Fold change	Adj. p value	Probe set/ Gene name	Fold change	Adj. p value	Probe set/ Gene name	Fold change	Adj. p value
FAM26F	2.60	0.001	HLA-C	9.66	<0.001	IFITM1	15.31	<0.001
FBXO6	2.53	0.002	HLA-C	4.61	<0.001	IFITM2	2.28	<0.001
FNIP1	2.17	0.019	HLA-C	9.10	<0.001	IFITM3	2.89	<0.001
FTSJD2	2.05	<0.001	HLA-C	8.50	<0.001	IGF1	1.80	0.007
GBP1	10.50	<0.001	HLA-E	3.00	<0.001	IL15	3.20	0.003
GBP1	8.49	<0.001	HLA-E	3.15	<0.001	IL18BP	2.80	<0.001
GBP1	7.23	<0.001	HLA-E	4.14	<0.001	IL20RB	2.67	<0.001
GBP1	1.85	0.014	HLA-F	5.42	<0.001	IL4I1	1.97	0.001
GBP2	3.10	0.003	HLA-F	5.45	<0.001	IL7	1.88	0.001
GBP2	3.33	0.001	HLA-G	4.18	<0.001	INHBA	2.26	0.046
GBP3	4.74	<0.001	HLA-G	5.33	<0.001	INHBE	2.74	0.008
GCH1	4.43	<0.001	HLA-G	6.17	<0.001	IRF1	2.47	<0.001
GCNT1	1.81	0.001	HLA-G	3.88	<0.001	IRF1	2.30	<0.001
GDF15	1.81	0.002	HLA-J	2.50	<0.001	IRF7	8.18	<0.001
GIMAP2	3.61	<0.001	IDO1	3.02	<0.001	IRF9	4.88	<0.001
GLRX	1.82	0.001	IFI16	3.92	<0.001	ISG15	34.81	<0.001
GMPR	5.21	<0.001	IFI16	5.05	<0.001	ISG20	10.01	<0.001
GNB4	2.28	<0.001	IFI16	4.38	<0.001	ISG20	9.06	<0.001
GRIA1	2.22	0.001	IFI27	230.11	<0.001	KIAA1755	3.00	<0.001
GRIA1	2.15	0.019	IFI30	3.46	<0.001	KMO	1.84	0.007
H1F0	2.21	<0.001	IFI35	18.98	<0.001	LAMP3	5.55	<0.001
HCP5	2.03	<0.001	IFI44	18.25	<0.001	LAP3	4.88	<0.001
HELZ2	5.22	<0.001	IFI44	10.18	<0.001	LGALS3BP	13.51	<0.001
HELZ2	1.95	0.001	IFI44L	5.38	<0.001	LGMN	1.86	<0.001
HERC5	56.94	<0.001	IFI6	49.89	<0.001	LNPEP	2.07	0.008
HERC6	43.41	<0.001	IFIH1	14.55	<0.001	LNPEP	1.90	0.005
HES4	3.43	<0.001	IFIH1	6.72	<0.001	LOC100132707	1.85	0.004
HIST1H2AC	2.25	<0.001	IFIH1	154.52	<0.001	LOC100310756	1.94	0.005
HIST1H2BD	1.83	0.003	IFIT1	66.60	<0.001	LOC100506274	2.28	0.002
HK2	2.07	<0.001	IFIT2	17.65	<0.001	LOC100507463	7.66	<0.001
HLA-A	5.09	<0.001	IFIT2	46.20	<0.001	LOC100507535	3.42	<0.001
HLA-A	5.27	<0.001	IFIT3	31.84	<0.001	LOC283501	1.90	0.001
HLA-B	6.43	<0.001	IFIT3	38.99	<0.001	LOC284561	2.00	0.002
HLA-B	7.81	<0.001	IFIT5	4.90	<0.001	LOC339260	2.08	0.004
HLA-B	6.26	<0.001	IFIT5	5.04	<0.001	LOC643401	1.86	0.003

Supplementary figure S3 (2)

Supplementary Table S1		500 pM Interferon beta						
Probe set/ Gene name	Fold change	Adj. p value	Probe set/ Gene name	Fold change	Adj. p value	Probe set/ Gene name	Fold change	Adj. p value
LY6E	2.76	<0.001	216565_x_at	4.23	<0.001	58916_at	2.83	<0.001
LYSMD2	2.32	0.002	217604_at	2.03	<0.001	NAMPT	2.10	0.011
MET	1.87	0.005	218280_x_at	2.12	<0.001	NAPA	1.81	0.009
MFNG	1.81	0.002	219545_at	1.81	0.004	NLRC5	7.17	<0.001
MICB	3.14	<0.001	220445_s_at	2.52	0.012	NMI	8.08	<0.001
MLKL	3.35	<0.001	221044_s_at	6.23	<0.001	NOD2	1.85	<0.001
MMP13	3.02	<0.001	222631_at	1.97	<0.001	NT5C3	2.96	<0.001
MOCOS	2.36	<0.001	224175_s_at	2.17	0.001	NT5E	1.89	0.023
MOV10	1.91	0.002	226040_at	2.48	<0.001	NT5E	2.07	<0.001
MT1E	2.19	0.001	226725_at	2.17	<0.001	OAS1	62.08	<0.001
MT1E	2.05	0.004	230233_at	1.94	0.006	OAS1	87.76	<0.001
MT1F	2.65	<0.001	230314_at	7.89	<0.001	OAS2	28.03	<0.001
MT1H	3.18	<0.001	232150_at	2.20	<0.001	OAS2	3.28	<0.001
MT1P2	2.80	<0.001	232375_at	5.46	0.001	OAS2	25.29	<0.001
MT1X	3.53	<0.001	232544_at	1.92	0.017	OAS3	13.26	<0.001
MT2A	3.42	<0.001	232615_at	2.01	0.003	OAS3	5.92	<0.001
MX1	90.51	<0.001	233050_at	2.71	<0.001	OASL	11.02	<0.001
MX2	19.74	<0.001	235157_at	8.00	<0.001	OASL	7.60	<0.001
MYD88	2.53	<0.001	235456_at	3.33	<0.001	OPTN	1.97	<0.001
MYL12A	1.86	0.005	235543_at	1.96	0.003	OR52K3P	3.45	<0.001
1552703_s_at	12.56	<0.001	235681_at	3.05	<0.001	OSMR	2.40	0.005
1561180_at	1.99	0.011	236692_at	1.84	0.003	PARP12	14.52	<0.001
1563075_s_at	2.81	0.010	237105_at	2.46	0.001	PARP14	13.20	<0.001
1563357_at	1.90	0.010	238743_at	2.67	0.002	PARP6	1.83	0.005
1566887_x_at	1.98	0.002	239587_at	21.50	<0.001	PARP9	15.41	<0.001
201601_x_at	13.90	<0.001	239979_at	5.04	<0.001	PARP9	3.80	<0.001
204820_s_at	3.89	<0.001	239988_at	1.92	0.004	PATL1	1.86	<0.001
205871_at	2.11	0.001	240382_at	2.55	0.001	PCGF5	2.07	0.001
208078_s_at	3.87	<0.001	241916_at	10.19	<0.001	PCGF5	2.08	<0.001
208523_x_at	1.82	0.015	241956_at	1.91	0.003	PCGF5	2.25	<0.001
209446_s_at	1.81	0.011	242667_at	2.00	0.002	PCGF5	2.48	0.004
214290_s_at	2.24	<0.001	242878_at	3.28	<0.001	PDZD2	2.13	0.001
214455_at	2.20	0.004	243271_at	4.45	<0.001	PHF11	4.01	<0.001
214808_at	2.67	0.005	243299_at	2.16	0.008	PHF11	2.06	0.001
214995_s_at	2.29	0.001	243576_at	2.90	0.002	PIK3AP1	6.28	<0.001

Supplementary figure S3 (3)

Supplementary Table S1		500 pM Interferon beta						
Probe set/ Gene name	Fold change	Adj. p value	Probe set/ Gene name	Fold change	Adj. p value	Probe set/ Gene name	Fold change	Adj. p value
PLA1A	2.30	<0.001	RNF213	3.37	<0.001	SP100	8.24	<0.001
PLEKHG4B	1.95	0.022	RSAD2	66.83	<0.001	SP100	9.77	<0.001
PLSCR1	7.95	<0.001	RSAD2	91.63	<0.001	SP100	15.37	<0.001
PLSCR1	8.47	<0.001	RTP4	12.42	<0.001	SP100	4.20	<0.001
PML	2.36	0.001	SAMD9	30.36	<0.001	SP100	16.16	<0.001
PML	1.82	0.002	SAMD9	149.90	<0.001	SP100	4.22	<0.001
PML	2.45	<0.001	SAMD9L	16.90	<0.001	SP110	8.78	<0.001
PML	2.40	<0.001	SAMD9L	9.08	<0.001	SP110	8.35	<0.001
PNPT1	4.66	<0.001	SAMD9L	18.79	<0.001	SP110	10.96	<0.001
PPM1K	3.24	<0.001	SAMHD1	19.66	<0.001	SP110	13.30	<0.001
PPM1K	3.75	<0.001	SAMHD1	20.08	<0.001	SP110	17.98	<0.001
PPM1K	4.44	<0.001	SAMHD1	13.97	<0.001	SP140L	3.40	<0.001
PPM1K	2.49	0.001	SAMHD1	12.76	<0.001	SPATS2L	2.32	0.002
PSMB8	12.04	<0.001	SAMHD1	11.87	<0.001	SPATS2L	2.20	<0.001
PSMB9	32.37	<0.001	SCARB2	1.90	<0.001	SPTBN5	1.98	0.002
PSME1	2.50	<0.001	SCARB2	1.91	0.002	SQRDL	3.17	<0.001
PSME2	2.50	<0.001	SCIN	1.90	0.011	STAT1	7.63	<0.001
RABGAP1L	1.98	0.001	SDPR	1.84	0.012	STAT1	15.93	<0.001
RABGAP1L	2.06	0.003	SECTM1	3.23	<0.001	STAT1	8.25	<0.001
RARRES3	4.11	<0.001	SELL	1.92	0.015	STAT1	13.60	<0.001
RASGRP3	2.82	0.001	SERPINB9	2.38	<0.001	STAT1	10.98	<0.001
RBCK1	2.09	<0.001	SERPING1	3.19	<0.001	STAT1	9.94	<0.001
RBM43	1.88	<0.001	SLC15A3	8.23	<0.001	STAT2	3.09	<0.001
RBM43	3.20	<0.001	SLC16A3	2.50	0.001	STC2	2.15	0.001
RET	2.53	<0.001	SLC16A3	2.49	0.005	STON2	2.65	<0.001
RHOC	2.03	0.008	SLC25A28	1.85	0.004	STON2	2.13	0.002
RIMBP2	1.95	0.002	SLC25A28	1.87	0.001	TAP1	18.37	<0.001
RIMS2	2.42	0.001	SLC2A12	1.81	0.010	TAP2	6.81	<0.001
RNF19B	2.04	0.001	SLC44A1	1.94	0.004	TAP2	2.06	0.001
RNF19B	2.14	0.006	SLC8A1	1.85	0.001	TAP2	12.13	<0.001
RNF213	7.63	<0.001	SLFN5	3.09	0.004	TAPBP	2.34	0.001
RNF213	7.80	<0.001	SLFN5	2.70	0.002	TAPBPL	2.19	0.019
RNF213	4.51	<0.001	SLFN5	2.25	<0.001	TAPBPL	2.06	<0.001
RNF213	7.91	<0.001	SLFN5	2.10	<0.001	TDRD7	13.17	<0.001
RNF213	6.64	<0.001	SMCHD1	1.87	0.002	TGFB2	1.98	0.003

Supplementary figure S3 (4)

Supplementary Table S1			500 pM Interferon beta					
Probe set/ Gene name	Fold change	Adj. p value	Probe set/ Gene name	Fold change	Adj. p value	Probe set/ Gene name	Fold change	Adj. p value
TGM2	1.92	0.003	USP18	21.96	<0.001	GLS	0.41	0.001
TLR3	11.21	<0.001	VDR	1.87	0.005	GLS	0.41	0.001
TMEM140	2.48	<0.001	VSIG10L	1.91	0.012	HSD17B14	0.50	0.020
TMEM62	2.15	0.001	WARS	3.26	<0.001	HSD17B14	0.42	0.002
TNFAIP6	2.11	<0.001	WARS	3.18	<0.001	HSD17B14	0.46	0.003
TNFAIP6	1.94	0.001	WDFY1	1.96	<0.001	ITGA8	0.54	0.004
TNFSF10	11.26	<0.001	XAF1	79.48	<0.001	KCNK2	0.53	0.013
TNFSF10	13.96	<0.001	XAF1	48.30	<0.001	KDM5B-AS1	0.54	<0.001
TNFSF10	13.23	<0.001	XAF1	4.33	<0.001	LINC00622	0.34	<0.001
TNFSF13B	6.05	<0.001	ZC3HAV1	4.46	<0.001	LINC00645	0.44	0.001
TNFSF13B	13.99	<0.001	ZC3HAV1	6.49	<0.001	LOC100507303	0.52	0.001
TPD52	1.90	0.032	ZC3HAV1	3.95	<0.001	LOC338620	0.51	0.001
TRANK1	2.47	<0.001	ZCCHC2	2.66	<0.001	LONRF2	0.23	<0.001
TREX1	2.32	<0.001	ZCCHC2	2.74	<0.001	LONRF2	0.38	0.001
TRIM14	2.53	0.002	ZNFX1	3.88	<0.001	LRPAP1	0.52	0.001
TRIM14	9.04	<0.001	ACO1	0.30	<0.001	LRRC3B	0.52	<0.001
TRIM21	3.71	<0.001	AGO1	0.39	<0.001	1556216_s_at	0.54	0.001
TRIM22	4.22	<0.001	AGTR1	0.51	0.001	1556306_at	0.37	<0.001
TRIM25	2.08	0.001	AGTR1	0.52	0.002	1558170_at	0.47	0.006
TRIM25	6.42	<0.001	ARHGAP28	0.55	0.002	1558605_at	0.44	<0.001
TRIM38	2.56	<0.001	ARRB1	0.52	0.004	1559116_s_at	0.49	0.001
TRIM38	2.90	<0.001	ATP5E	0.55	0.004	1561511_at	0.52	<0.001
TRIM38	3.17	0.001	ATP6	0.52	0.001	210292_s_at	0.43	<0.001
TRIM38	2.15	0.003	BICD1	0.53	0.002	213486_at	0.48	<0.001
TRIM5	1.93	0.001	C7orf41	0.32	<0.001	215128_at	0.41	0.001
TRIM5	1.83	0.024	CDH8	0.55	0.001	215306_at	0.45	<0.001
TRIM56	2.89	<0.001	CLN8	0.37	0.002	223707_at	0.33	<0.001
TRIM6	2.37	<0.001	CLN8	0.24	0.001	224763_at	0.52	0.001
TRIM69	3.79	<0.001	DANCR	0.52	<0.001	226587_at	0.49	0.001
TXNIP	2.68	<0.001	EDN3	0.53	0.004	226591_at	0.43	0.004
TXNIP	2.42	<0.001	EIF3L	0.54	0.001	228370_at	0.48	<0.001
TXNIP	2.59	<0.001	FABP4	0.47	0.013	228740_at	0.24	<0.001
UBA7	4.02	<0.001	FAM172A	0.55	0.002	229544_at	0.36	<0.001
UBA7	4.78	<0.001	GABRR1	0.46	<0.001	230248_x_at	0.44	0.001
UBE2L6	5.11	<0.001	GLS	0.45	0.001	231047_at	0.53	0.008

Supplementary figure S3 (5)

Supplementary Table S1			500 pM Interferon beta		
Probe set/ Gene name	Fold change	Adj. p value	Probe set/ Gene name	Fold change	Adj. p value
235679_at	0.50	0.002	STRBP	0.32	<0.001
236032_at	0.54	0.002	THEMIS	0.46	0.003
236330_at	0.43	<0.001	TLE2	0.55	<0.001
237390_at	0.45	<0.001	TLL2	0.52	<0.001
237419_at	0.47	0.002	TNFRSF10D	0.39	0.002
238824_at	0.43	0.001	TNFRSF10D	0.20	<0.001
241156_at	0.49	0.001			
241407_at	0.52	0.031			
241772_at	0.54	0.002			
241873_at	0.50	0.001			
242523_at	0.56	0.002			
242928_at	0.51	0.001			
243918_at	0.43	0.002			
NAA38	0.54	0.003			
NANOS1	0.52	0.002			
NFYA	0.45	0.004			
NFYA	0.24	<0.001			
OTUD3	0.50	0.002			
PAR5	0.33	0.005			
PCBD2	0.53	<0.001			
PCDH20	0.49	<0.001			
PCDP1	0.52	0.014			
PDE1A	0.54	0.009			
PDE1A	0.50	0.001			
PGAP1	0.55	0.003			
PI15	0.51	<0.001			
PTGFR	0.50	0.002			
PTGFR	0.39	0.007			
RPS15A	0.54	0.001			
SCN4B	0.51	0.002			
SCN7A	0.52	<0.001			
SLC1A6	0.52	0.007			
SMEK2	0.51	0.002			
SPAG16	0.54	0.005			
STEAP4	0.48	0.003			

Supplementary figure S3 (6)

Fig S2: NCC were exposed for 48 h to IFNβ or to the respective solvent control. Then cells were harvested, and total RNA was extracted. Analysis was then performed using Affymetrix chip-based DNA microarray (Human genome U133 plus 2.0 arrays) with all standard normalization quality control procedures (see Materials and Methods). The differentially expressed probe sets for each compound, including fold changes and p values of the limma t test are listed.

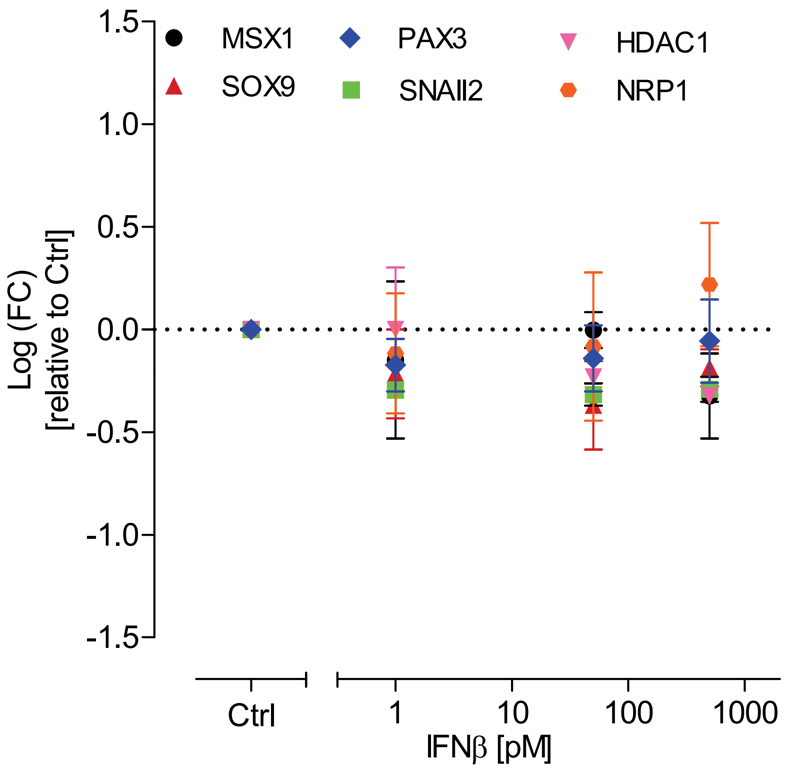

Supplementary figure S4

Fig S3: NCC were exposed to IFNβ for 48 h at the indicated concentrations. Then cells were harvested, and total RNA was extracted and retro-transcribed. Effects on selected mRNAs were evaluated by qPCR. Expression levels were normalized against the housekeeping gene, GAPDH, and expressed relative to those of untreated cells (Ctrl). Data are from three independent experiments. Error bars indicate standard deviations (SD).

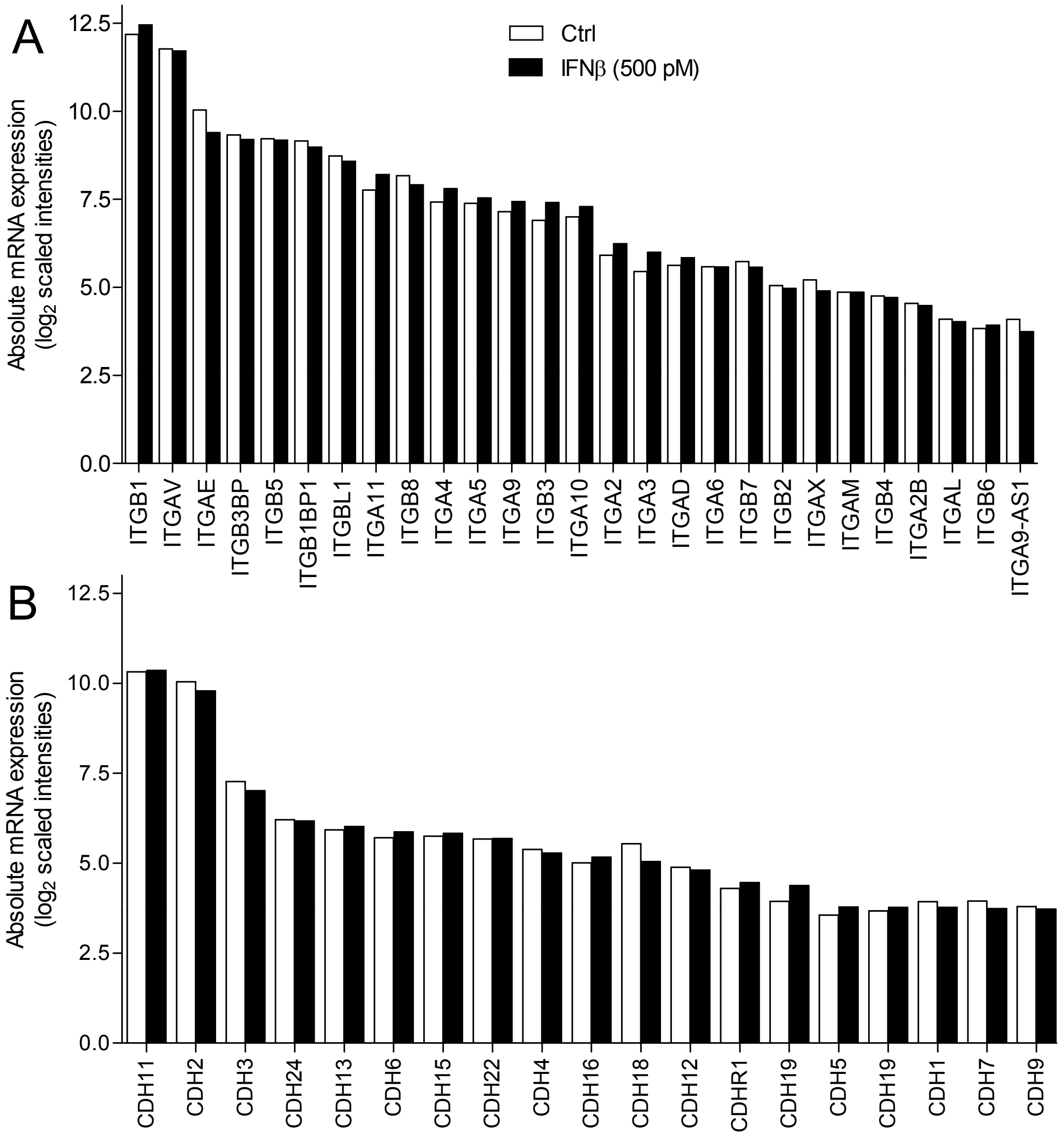

Supplementary figure S5

Fig S4: NCC were exposed for 48 h to IFNβ or to the respective solvent control. Then cells were harvested, and total RNA was extracted. Analysis was then performed using Affymetrix chip-based DNA microarray (Human genome U133 plus 2.0 arrays) with all standard normalization quality control procedures (see Materials and Methods). **A** The absolute mRNA expression of integrins, and **B** cadherins was averaged among probesets and reported on the y axis. Data are fluorescence intensities of the average probe sets on a log_2 scale. Genes that are not expressed had a fluorescence level of 4-5.

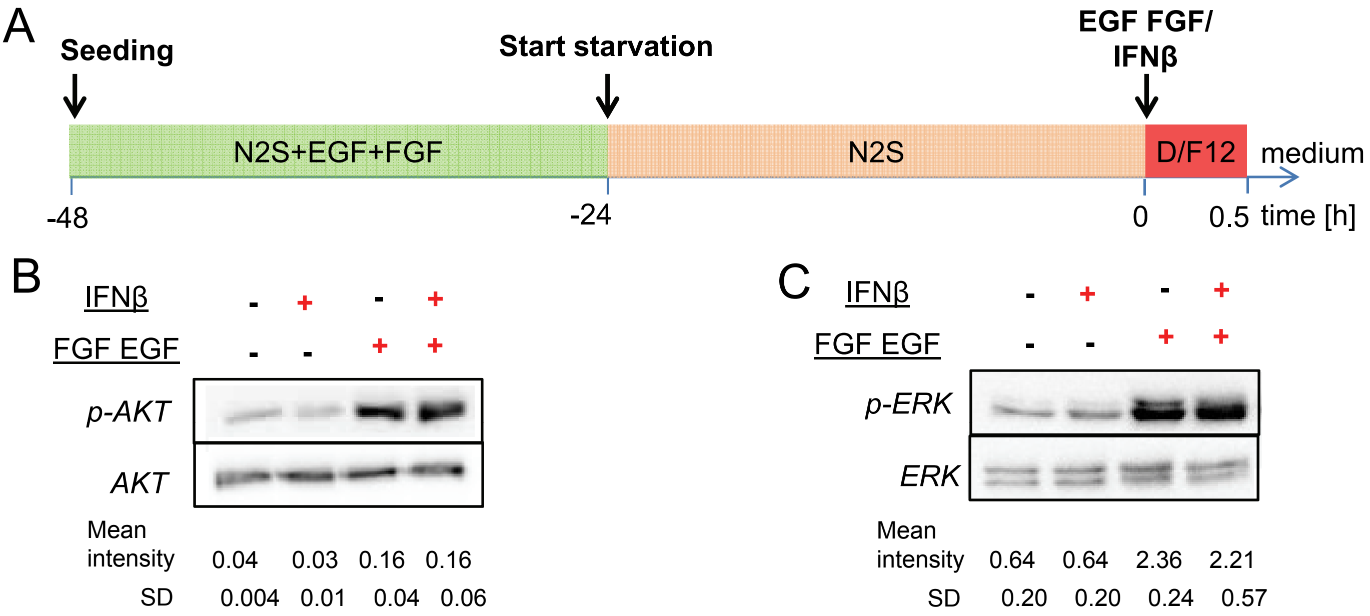

Supplementary figure S6

Fig S5: A NCC were seeded and incubated for 24 h in N2S medium in the presence of the cytokines EGF (20 ng/ml) and FGF (20 ng/ml) (see Materials and Methods for NCC maintenance). Next, NCC were starved for 24 h in N2S medium in absence of cytokines. NCC were then exposed for 0.5 h to the growth factors (EGF+FGF) diluted in DMEM/F12 medium, in order to trigger known signaling cascades. The last step was examined in the presence of absence of IFNβ (500 pM) during the growth factor stimulation. After this, cells were harvested, and total protein samples prepared. **B, C** The amount of phosphorylated AKT (p-AKT) or phosphorylated ERK (p-ERK) was measured by western blot analysis (representative blots are shown). The mean intensity of each band normalized to the respective not phosphorylated forms (AKT or ERK) ± SD is reported below each condition (n = 3)
